# Helically shaped cation receptor: design, synthesis, characterisation and first application to ion transport†

**DOI:** 10.1039/d0ra05519k

**Published:** 2020-08-27

**Authors:** Hamza Boufroura, Romain Plais, Salomé Poyer, Anne Gaucher, Jérome Marrot, Gilles Clavier, François-Xavier Legrand, Cécile Huin, Philippe Guégan, Damien Prim, Jean-Yves Salpin

**Affiliations:** Université Paris-Saclay, UVSQ, CNRS, UMR 8180, Institut Lavoisier de Versailles 78000 Versailles France damien.prim@uvsq.fr; Université Paris-Saclay, CNR, Univ Evry, LAMBE 91025 Evry France; CY Cergy Paris Université, CNRS, LAMBE 95000 Cergy France; Université Paris-Saclay, CNRS, ENS Paris-Saclay, PPSM 94235 Cachan France; Université Paris-Saclay, CNRS, Institut Galien Paris-Saclay 92290 Châtenay-Malabry France; Sorbonne Université, CNRS, Institut Parisien de Chimie Moléculaire, Equipe Chimie des Polymères 4 Place Jussieu 75005 Paris France; Université Paris-Saclay, Université d’Evry Val d’Essonne 91025 Evry France

## Abstract

The methyl ester of 8-oxo-8*H*-indeno[2′,1′:7,8]naphtho[1,2-*b*]thiophene-2-carboxylic acid (1) and its corresponding PEGylated ester were synthesised and fully characterised. X-ray diffraction studies on (1) confirmed the helical structure of the receptor and that it is self-assembled into layers by π–π interactions. An in-depth study by DFT calculations and MS experiments (ESI-MS, MS/MS, IMRPD and ESI-IMS-MS) was carried out between (1) and the physiological cation K^+^. The formation of supramolecular complexes between (1) and K^+^ with different stoichiometries was demonstrated and the cation K^+^ preferentially interacts with the oxygen atoms of the carbonyl bond of the ketone and ester groups and the sulphur atom of the heterocycle. The ability of the two synthesized aromatic architectures to transport ions across a model lipid membrane has been studied by electrophysiology experiments. The formation of pores was observed, even at nanomolar concentrations. Since the PEGylated molecule showed more regular pore definitions than the hydrophobic molecule, the introduction of a polar hydrophilic chain made it possible to control the orientation of the aromatic architectures within the membrane.

## Abstract 2

An helicene-like receptor is able to transport K^+^ across lipid membrane.

## Graphical abstract

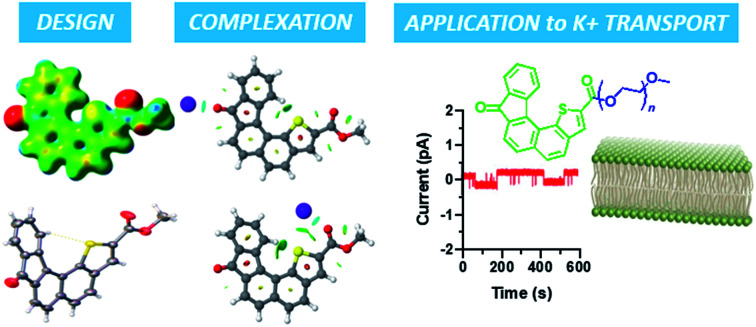

## Introduction

Transport of ions across cell membranes is a crucial biological process the misregulation of which causes severe diseases currently known as channelopathies.^1^ Recovery of regulation processes requires a deep understanding of the mechanisms that take place in natural ion channels. One way to gain information is the development of new artificial molecular models mimicking biological functions involved in ion transport.^2^ In general, synthetic architectures able to act as ion carriers can be split into two main families: preformed cyclic scaffolds and self-assembling in the lipidic bilayer.^3^ In both cases, ion transportation is mediated by weak interactions such as electrostatic and/or surface interactions. It is now admitted that the cation–π interaction is a major non covalent binding interaction for ion transport and more generally for biological recognition.^4–6^ Oxygen and sulphur often serve as coordinating atoms in a number of σ-donor and π-donor polar functional groups to bind alkali metal cations.^7–9^ Cation–π interactions between metal cations such as Na^+^, K^+^, Mg^2+^ or Ca^2+^ and aromatic systems also appear crucial in biological systems,^4,10,11^ contributing to the stabilization of particular conformations of proteins or the preservation of the charge balance during enzymatic catalytic processes^12,13^ for example. In this context, cation–π interactions with planar fused polyaromatic systems^14–16^ or polyaromatic macrocycles, such as calixarenes^17,18^ and pillar[*n*]arenes,^19–21^ and also more flexible *para*-oligophenylenes,^22–24^ have been theoretically and experimentally studied. Those molecular systems proved to be able to insert into a lipid bilayer facilitating ion transport across the membrane through cation–π interactions.^16,22–26^ The combination of hydrophobic aromatic rings and hydrophilic residues into a single molecule has been explored by several groups at both theoretical and experimental levels, evidencing the deep impact of the molecular topology on complexation properties using a template or promoting synergistic cation–π interactions.^12,27^

**Fig. 1:**
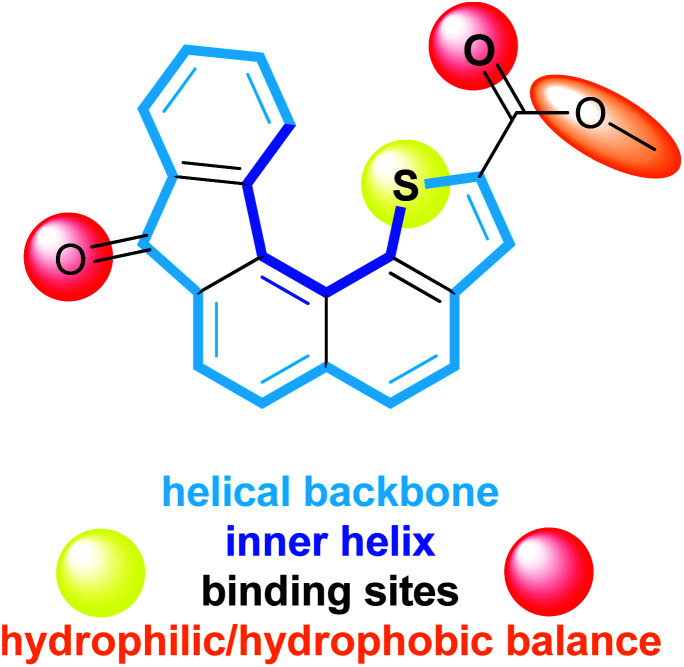
Characteristic structural features of receptor 1.

In order to gain further information about alkali metal cation–π interactions in 3D molecular architectures, we have developed a new synthetic receptor. The design of the receptor 1 (Fig. 1) is based on low molecular weight helical organic molecule. This helicene derivative comprises five fused rings which confer to the overall aromatic core a helical shape, a thiophene ring displaying the sulphur atom at the inner helix and two functional groups, namely a ketone and a carboxylic ester. The characteristic structural features of receptor 1 display several assets including: (i) a controlled and modulable non planar aromatic core, (ii) functional groups allowing electrostatic interactions and hydrophilic/hydrophobic balance, (iii) a direct proximity between the thiophene sulphur atom and the ester fragment favourable to cation complexation, (iv) a capped/protected potential binding site embedded into the inner helix.

In this paper, we report a combined theoretical and experimental study of the K^+^ complexation by receptor 1 and its polyethylene glycol (PEG) derivative 3 (*vide infra*). The intrinsic interactions occurring at the molecular level between metal ions and receptor 1 was first carried out by mass spectrometry and density function theory calculations. The ability of 1 and 3 to insert into lipid membrane and to transport K^+^ across membranes was notably also explored by mean of black lipid membrane (BLM) experiments.

## Results and discussion

### Synthesis and solid-state characterization

**Scheme 1:**
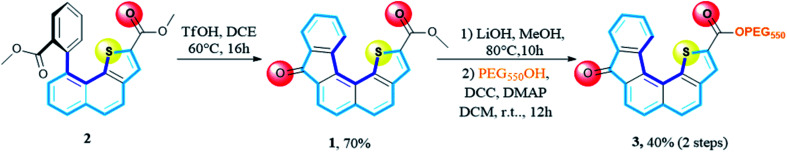
New set of cyclization conditions and modulation of the hydrophilic/hydrophobic balance.

The synthesis of receptor 1 is based on a recent report of one of us.^28^ The synthetic sequence involved a Friedel–Crafts type reaction from ester precursor 2 using polyphosphoric acid or phosphorus pentoxide heated at 100 °C. The expected target 1 was obtained, with average yields due to the presence of several side products rendering the purification step tedious. In the present context, we set a new experimental procedure by using trifluoromethanesulfonic acid in DCE, heated at 60 °C for 16 hours. Under these conditions the desired receptor 1 was cleanly obtained in a fair 70% yield without the presence of byproducts (Scheme 1).

Bearing in mind the evaluation of receptor 1 transport ability across membranes, we also examined the hydrophilic/hydrophobic balance by modifying the ester residue. To this end, we envisioned the installation of a polyethylene glycol (PEG) chain instead of the starting methyl group. Saponification of the methyl ester group using LiOH, in MeOH at 80 °C until completion (10 h), followed by coupling using mPEG_550_ in the presence of DCC and DMAP in methylene chloride at room temperature for 12 hours, account for the two-step sequence leading to receptor 3 with an overall yield of 40%.

**Fig. 2:**
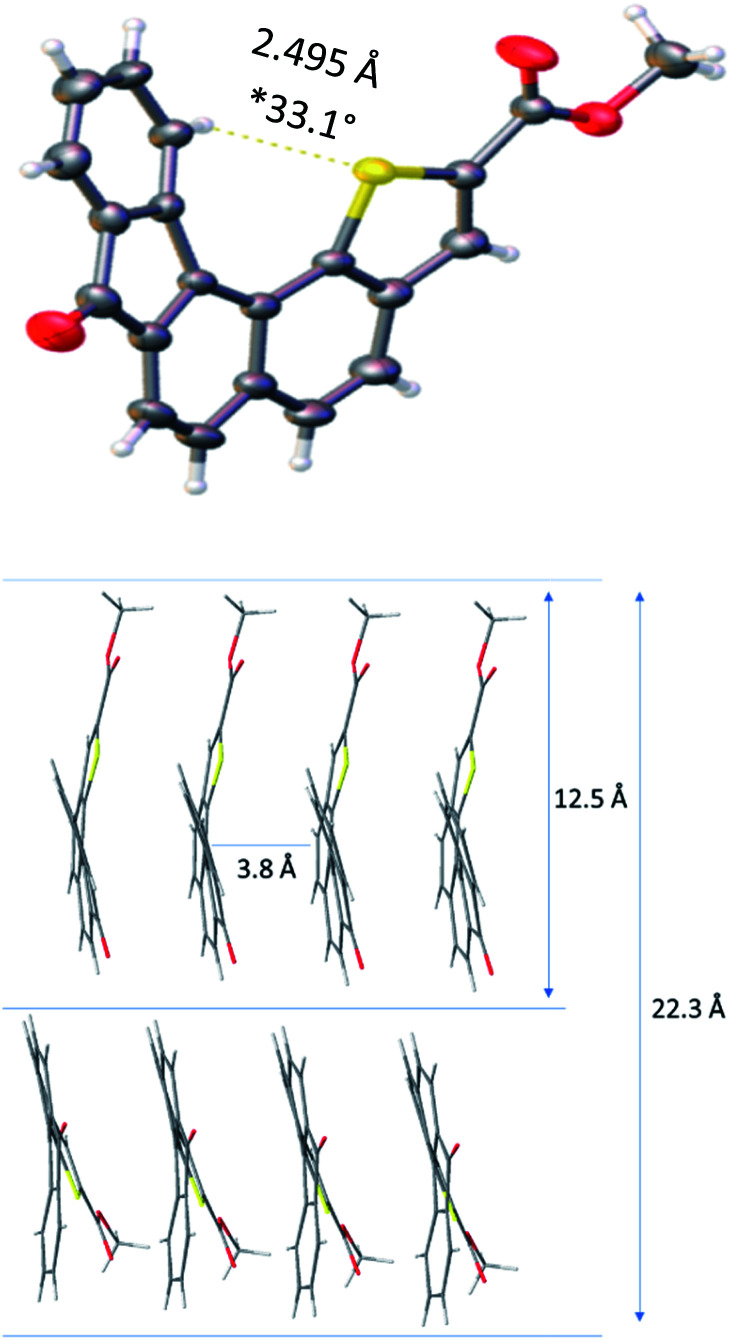
X-ray diffraction structure and key spatial arrangement (*dihedral angle is measured between the two extreme aromatic rings).

Slow evaporation of a solution of 1 in methylene chloride afforded suitable crystals for X-ray analysis and allowed gaining structural information in the solid state. The resolved structure evidenced a well-organized self-assembly of single units and highlighted that the presence of a sulphur atom located at the inner helix of the architecture induced a C–H⋯S interaction which contributes to helical shape of receptor 1 (Fig. 2). The distance between the methyl group of the ester moiety and the carbonyl function of the fluorenone scaffold was found to be 12.5 Å and appeared to be the longest one measured on the monomer.

Moreover, the solid state showed the formation of crystal packing in which monomers self-assemble into layers. Indeed, each molecule interacts with one another through a face-to-face π–π interaction with a 3.84 Å measured distance. This allowed us to determine the distance between two units, with methyl groups in the opposite direction, which was found to be 22.3 Å. The latter distance implemented by the PEG chain length might contribute to reach the common 37 Å length^29^ required to fully cross the lipid bilayer and balance the hydrophobicity of receptor 1.^30,31^

Taking into account all these observations we envisioned that these helicene derivatives could possibly present ion-transporting properties across the lipid membrane.

### Computational study

**Fig. 3:**
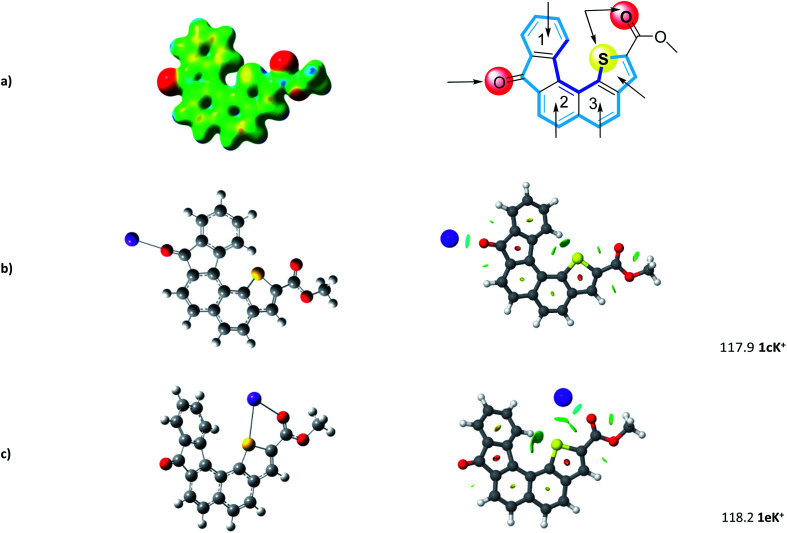
(a) Computed structure of 1 (ESP) and calculated binding sites. (b) and (c) Most favorable 1–K^+^ structures and corresponding NCI (non-covalent interaction) plot. Binding energies are reported in kJ mol^−1^.

The geometry of the receptor 1 was first studied in order to evaluate its ability to host an alkali cation such as K^+^. The optimized structure at the M06-2X/6-311++G(d,p) level of theory is represented in Fig. 3. The calculated quadrupole moment of 1 (−155 B) confirmed the tendency of 1 to preferentially interact with cations in complete agreement with recent literature.^32^ From ESP (electrostatic potential surfaces) map of receptor 1, we anticipated that interaction between K^+^ and the helical carbon backbone will only occur to a minor extent. In contrast, the presence of both carbonyl group and sulfur atom contributes to two well-defined electronically rich areas suitable for complexation of K^+^. As already reported,^27^ the thiophene sulphur atom and the ester carbonyl group display a *syn* conformation which constitutes a favourable complexation pattern.

Further step was the determination of most propitious binding sites and of binding energies (BE) at the M06-2X/6-311++G(2df,2pd)//M06-2X/6-31+G(d,p) level. To this end, potential binding sites including three six-membered rings, the thiophene moiety, but also the carbonyl and the combination sulfur atom-carbonyl group, were evaluated and their binding energies (BE) determined (see Fig. S1†). As foreseen, potential K^+^ complexes arising from cation–π interaction at aromatic sites display lower BE ranging from 78 to 102 kJ mol^−1^.

In complete agreement with ESP map, 1cK^+^ displays a BE of 117.9 kJ mol^−1^. Our systematic complexation study evidenced that 1eK^+^ exhibits a similar binding energy of 118.2 kJ mol^−1^. Note the computed binding energies vary only slightly when increasing the level of calculation (see ESI†). NCI plot confirms on one hand the strong interaction of K^+^ with the C=O group, in 1cK^+^, and on the other hand the presence of S–K^+^ interaction which stabilize the K^+^ complex for 1eK^+^. Interestingly, no additional contribution from the π-cloud of phenyl ring 1 was determined neither in 1cK^+^, nor in 1eK^+^.

### Experiments in solution

Interaction between 1 and potassium cation was first examined in solution by means of ^1^H NMR titrations. Unfortunately, if 1 was fully soluble in CDCl_3_ and CD_2_Cl_2_ at 1 × 10^−3^ mol L^−1^, classical potassium salts (KBF_4_, KPF_6_) and KTPBP (potassium tetrakis[3,5-bis(trifluoromethyl)phenyl]borate) remained unsoluble or only poorly soluble in solvent such as CDCl_3_, CD_2_Cl_2_ or combinations of CDCl_3_/acetonitrile or CD_2_Cl_2_/acetone in various radii. This issue precludes thus observations of any modifications of the ^1^H NMR spectra fingerprint characteristic of potential interactions between aromatic protons and potassium cation.

**Fig. 4:**
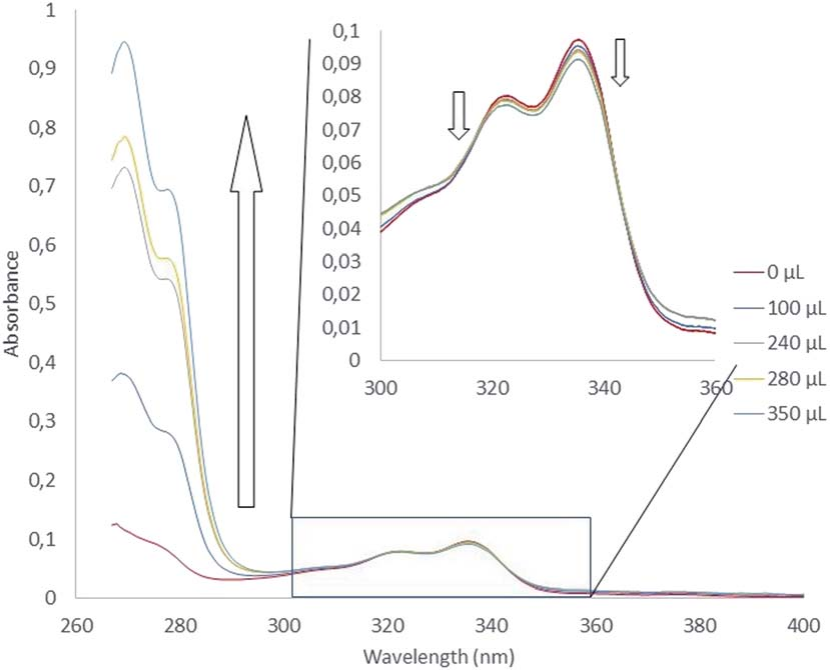
Absorption spectra measured during titration of 1 by KTBPB from 0 to 63 equivalents of potassium cation ([1] = 3,6 μM and [KTBPB] = 1,63 mM).

We next turned our attention to absorption spectroscopy. Absorption spectroscopy titration experiments are reported in Fig. 4. Absorption spectrum of 1 displayed four absorption maxima at 268, 306, 322 and 336 nm (Fig. S2†). A TDDFT calculation (PBE1PBE/6-311++g(d,p) level) allowed assigning these ban four bands to π → π* transitions highly delocalized over the helical architecture (Fig. S3–S5†). In THF, the iterative additions of potassium equivalents lead to substantial modifications of the spectra. Strong hyperchromic shift of the transitions between 260 and 280 nm as well as slight hypochromic shifts of bands centered on 320 and 340 nm were observed.

Global spectral analysis with the SPECFIT software enabled the determination of the association constant. Formation of a 1 : 1 host : guest complex was assessed with an association constant of 1242 L mol^−1^. Noteworthy, 2 : 1 binding model was also tested but did not converge and fit with our experimental results.

### Mass spectroscopy (MS) experiments

Prior to BLM experiments, a mass spectrometry study has been carried out in order to get information about the intrinsic interaction of 1 with K^+^. Indeed, MS experiments may be useful to give an insight about the interactions occurring at the molecular level between metal ions and ligands, presently the helicene structure 1. A series of experiments was carried out in order to determine the number of helicene units which can interact with the metal ion. In addition, ion mobility mass spectrometry (IM-MS) experiments were also conducted to evaluate the three-dimensional conformation of 1 with K^+^. This technique allows the separation of gaseous ions according to their charge and shape and can also allow the determination of collision cross sections (CCS), which, by comparison with simulations, can give indications about the three-dimensional conformation of the ions studied.

### ESI-MS(/MS) analysis

Experiments were carried out using different mass analyzers in order to determine the number of helicene units which can interact with the potassium cation. For this purpose, a quadrupole ion trap mass spectrometer (QIT), a hybrid quadrupole-time of flight (Q-TOF) and a triple-quadrupole (QqQ), all equipped with electrospray sources, were used (experimental details can be found in the ESI†). The comparison of different technologies and generations of mass spectrometers allows our results to be more comprehensive as the formation and observation of adducts may rely to the combination of ionization source/interface/MS analyzers used.^33^

The analysis of the receptor helicene-like 1 was first carried out without potassium. To this end, a methanolic solution of 1 was prepared and directly introduced in the mass spectrometer. The prominent species observed under ESI condition is the formation of the protonated species [1 + H]^+^ detected at *m*/*z* 345 (Fig S6†).

Singly charged multimers from di- to tetramers depending on the instrument used were observed around residual sodium cations under these conditions. The sodium may originate either from solvent, vials or sample 1, or is simply present in trace amounts in the mass spectrometers. From these experiments, protonated dimer [(1)_2_ + H]^+^ was not observed at the expected *m*/*z* 689. This suggests that this particular dimer is less prone to be formed than the sodiated dimer, even when using a protic solvent such as methanol.

The [1 + H]^+^ ion at *m*/*z* 345 was then selected in order to record its collision induced dissociation (CID) spectrum in order to get some insights about the possible protonation site(s). CID activation by collision with helium resulted in the spectrum displayed in Fig S7a,† which shows three main fragmentation channels involving the ester function, namely loss of methanol (*m*/*z* 313), carbon dioxide (*m*/*z* 301), and a methylformiate moiety (*m*/*z* 285). Unfortunately, these losses can be explained by charge remote mechanisms and do not allow the localization of the proton on the structure 1. Deuterium exchange experiments confirm these fragmentation mechanisms, as no losses of D instead of H were observed (Fig. S7b†).

The analysis of a mixture of 1 in a KCl solution was then carried out in order to determine the number of helicene units able to interact with K^+^ in the gas phase, and to obtain some structural information about the [(1)_*n*_ + K]^+^ complexes. For the sake of comparison, MS conditions have to be as close as possible to BLM experiments (*vide infra*). For this purpose, optimization of the various parameters of the instruments, concentrations and helicene/KCl molar ratios was carried out to reflect standard BLM experiment conditions. It is worth mentioning that the KCl concentrations used to perform the BLM experiments are usually too high for typical mass spectrometry conditions, and could therefore damage the instrument.

**Fig. 5:**
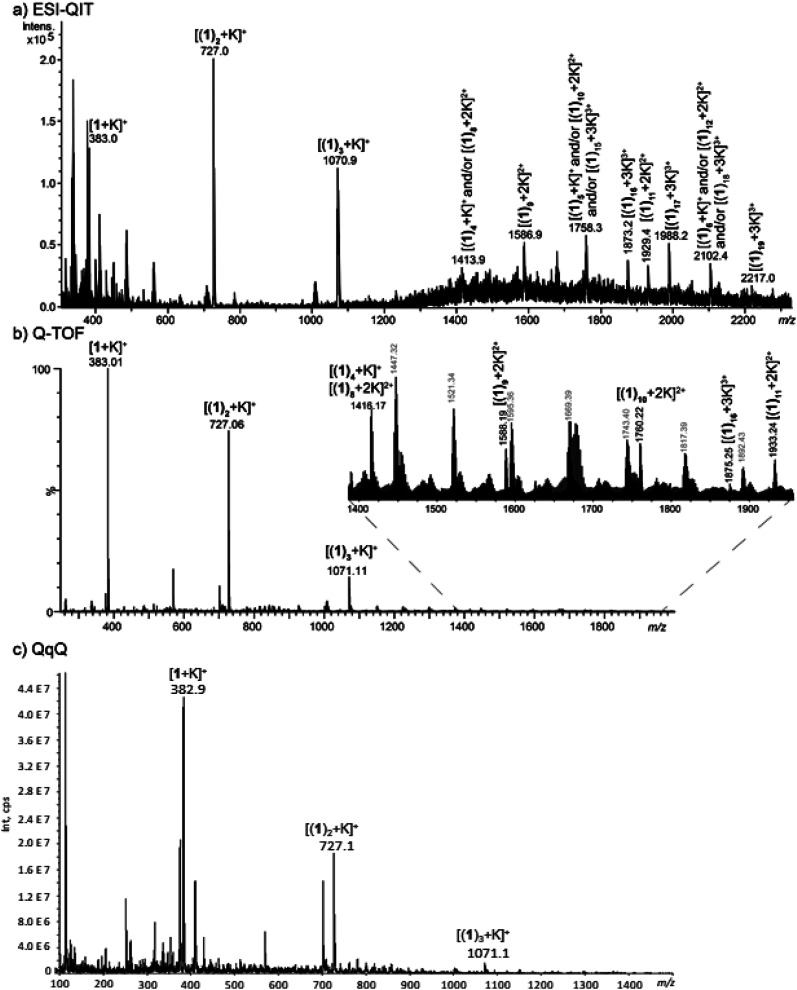
MS spectra obtained from a methanolic solution of KCl at 1 mM and 1 at 150 μM analysed (a) a QIT (b) a Q-TOF and (c) a QqQ mass spectrometer.

We first increased the concentration of KCl for a fixed concentration of 1 at 30 μmol L^−1^. The solvent used was methanol. We tried to use water as solvent, but with high KCl concentration, it led to the formation of potassium chloride clusters instead of complexes with 1. Formation of potassium adducts with 1 is already observed with a KCl concentration of 0.3 μM but competes with the formation of sodium adducts present in traces amounts in the instruments. By contrast, sodium cations interacting with 1 are no longer observed from mixtures of KCl concentration above 300 μM. The highest concentration value used before the onset KCl clusters (which are likely to be superimposed over or to replace multimeric [(1)_*n*_ + K]^+^) is 1 mM. We used this KCl concentration for our experiments in order to be as close as possible from BLM experimental conditions. In addition, the concentration in 1 was increased at 150 μmol L^−1^ to form larger multimers. In these conditions, formation of [(1)_*n*_ + K]^+^ (*n* = 1–3) is clearly overwhelming. However, we could observe from 1 to 19 helicenes 1 complexed to one K^+^ with the instrument allowing the milder source conditions (Fig. 5a). Note that MS signal at for example *m*/*z* 1758.3 can correspond either to singly-charged [(1)_5_ + K]^+^, doubly-charged [(1)_10_ + 2K]^2+^ or either triply-charged [(1)_15_ + 3K]^3+^ multimers. Charge state assignment can only be achieved by high resolution mass spectrometry. Presently, among the instruments used, only the Q-TOF offers a resolution high enough to identify the charge state of the multimer at a given *m*/*z* value. By combining Q-TOF and QIT experiments, we unambiguously observed singly charged species from [(1)_1_ + K]^+^ at *m*/*z* 383.0 to [(1)_4_ + K]^+^ at *m*/*z* 1416.2, doubly-charged multimers from [(1)_8_ + 2K]^2+^ at *m*/*z* 1416.2 to [(1)_11_ + 2K]^2+^ at *m*/*z* 1933.2, and triply-charged ions from [(1)_16_ + 3K]^3+^ at *m*/*z* 1875.3 to [(1)_19_ + 3K]^3+^ at *m*/*z* 2217.0 (Fig. 5b). Note that this multimeric distribution depends on the MS instrument. In summary, even if the three instruments presently used lead to similar results, that is overwhelming [(1)_*n*_ + K]^+^ ions, larger multimers could possibly be observed on different MS instruments. MS/MS experiments were performed in order to characterize the structure of the multimeric [(1)_*n*_ + K]^+^ species. For *n* = 1, 2 and 3, the loss of monomer(s) was exclusively observed starting from the precursor ion, and is in complete agreement with the interaction of K^+^ with several intact molecules of 1. The [1 + K]^+^ ion is very intense and recording its MS/MS spectrum is straightforward. However, this spectrum is not conclusive as it exhibits a prominent fragment at *m*/*z* 39 corresponding to K^+^ ions (Fig. S8†). Globally, these spectra do not give a detailed information about the actual structure of these ions. Notably, these experiments cannot decipher between a structure in which K^+^ interacts directly with several helicenes, and for example a structure in which K^+^ interacts with one molecule of helicene, the remaining helicenes self-assembling like in solid state (*vide supra*) through π-stacking.

### IRMPD experiments

In order to get further insight about the structure of the [1 + K]^+^ complex, we used IRMPD action spectroscopy in the fingerprint region. Experimental IRMPD spectra were recorded using the beamline of the free electron laser (FEL) of the Centre Laser Infrarouge d'Orsay (CLIO), which is coupled to a modified Bruker Esquire 3000+ 3D quadrupole ion trap.^34–36^

**Fig. 6:**
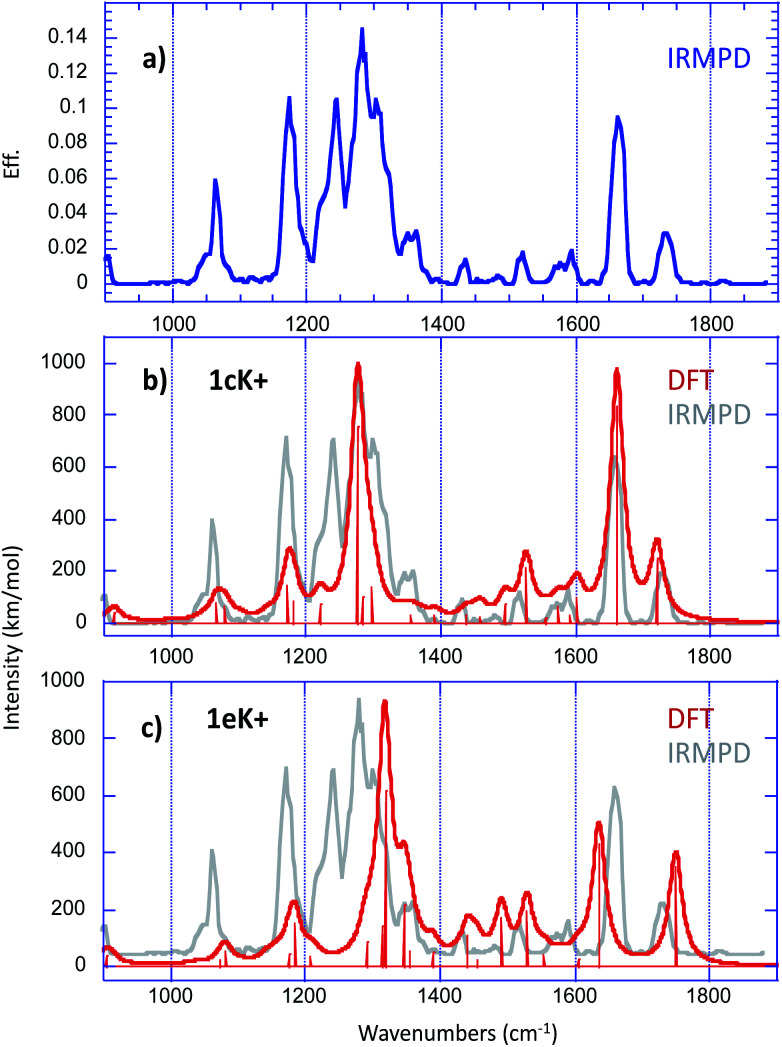
(a) Experimental IRMPD spectrum recorded in the fingerprint region for the [1 + K]^+^ complex. Overlay of experimental (in grey) and theoretical (in red) harmonic vibrational spectra computed at the B3LYP/6-31+G(d,p) level for (b) 1cK^+^ and (c) 1eK^+^ (scaling factor 0.974), convoluted with a 15 cm^−1^ Lorentzian function.

The IRMPD spectrum obtained for the [1 + K]^+^ ion is given in Fig. 6a.

The only photo-fragment observed during these experiments was the potassium cation. The experimental spectrum exhibits numerous absorptions with various intensities, which suggests that the [1 + K]^+^ complex may correspond to a mixture of different structures under our electrospray conditions.

Structural assignment by IRMPD is based on the comparison of the experimental trace with the spectra computed for the low energy-lying structures. Fig. 6b and c present the comparison with the vibrational spectra computed for the two most stable forms 1cK^+^ and 1eK^+^, which are practically degenerate. For a better comparison with the experimental IRMPD signal, computed spectra were convoluted with a 15 cm^−1^ Lorentzian function. The agreement with experiment turned to be slightly better when using the B3LYP functional than when using M06-2X (Fig. S9 and S10†). Several recent studies have already reported the good performances of the hybrid B3LYP functional in the context of the comparison between experimental IRMPD traces and computed harmonic vibrational spectra (this trend has been already reported previously in several reports^37,38^). Consequently, we rather chose the B3LYP functional. A scaling factor value of 0.974 has been applied in the 900–1800 cm^−1^ frequency range. Examination of Fig. 6 shows that, if one excepts the sharp feature measured at 1243 cm^−1^, all the experimental vibrations can be interpreted by the two computed spectra. Notably, the absorptions detected between 1400 and 1800 wavenumbers are nicely reproduced by the spectrum computed for 1cK^+^, and more particularly the bands observed experimentally at 1661 and 1736 cm^−1^, which can be attributed to the C=O stretches of the interacting and free carbonyl, respectively. The spectrum of 1cK^+^ also correctly reproduces the experimental features detected at 1064 and 1175 cm^−1^, and the most intense absorption at 1281 cm^−1^, which can be attributed to the C–O stretch of the ester function. On the other hand, the shoulder on the blue side of the most intense signal is well reproduced by the band computed at 1318 cm^−1^ for 1eK^+^ (the C–O stretch of the ester function). A good agreement is also observed with 1eK^+^ for the signal detected experimentally at 1361 cm^−1^. IRMPD data therefore suggest that a mixture of at least two forms may be generated under ESI conditions, and that these two forms are associated with the interaction of the potassium with a carbonyl group, either alone (1cK^+^) or in a bidentate scheme with sulphur atom (1eK^+^). Note also that the two C=O stretches observed experimentally cannot be reproduced computationally by the structures involving only an interaction with an aromatic ring, because in that case one the C=O stretch is no red shifted by the interaction with the alkali cation. Consequently, such a binding scheme can be reasonably discarded for the [1 + K]^+^ complex. The agreement is also not so good with 1aK^+^ and 1bK^+^. This is consistent with the fact that these two forms are sensibly less stable and should not be generated based on thermodynamical criteria.

Unlike MS/MS experiments, ion mobility spectrometry experiments may provide direct information about the tridimensional shape of this particular complex. Presently, the first information obtained from IMS-MS experiments is [(1)_*n*_ + K]^+^ ions (*n* = 1–3) exhibit a single IMS signal. This would mean that for [1 + K]^+^, either only one gas-phase conformation is observed, or that several conformations with similar collision cross section (CCS) co-exist. Indeed, the IMS resolution is relatively low and ions exhibiting different composition and conformations may have the same CCS. For both the [(1)_2_ + K]^+^ and [(1)_3_ + K]^+^ ions, the thinness of the peaks observed on the mobilograms suggests a single type of organization of 1 around K^+^. Larger peak widths are usually observed for multimers as different organization around the cation can be observed, resulting in an increase of conformational variations.^39^

In order to deduce a tridimensional structure of [1 + K]^+^ from ion mobility experiments, the IMS signal can be converted into CCS values. This implies first to perform the CCS calibration of the ion mobility cell, and the experimental CCS values can then be subsequently compared to calculated CCSs. The latters are estimated by using the modified N_2_ parameterized version of the MOBCAL software, which converts cartesian coordinates into CCS values. M06-2X calculations performed on the [1 + K]^+^ ion were presently used to estimate the theoretical CCS values (Table 1), to evaluate the three-dimensional conformation of 1 with K^+^. As the experimental CCS value is obtained from IMS cell calibration, error of 3% can generally be observed between CCS_exp_ and CCS_theo_.^40^ Consequently, the CCS values obtained for the 1dK^+^, 1eK^+^ and 1fK^+^ structures are in excellent agreement with the experimental value measured. On the other hand, no distinction between these three different conformations can be achieved experimentally. Although IRMPD experiments undoubtedly confirmed the presence of 1cK^+^, comparison of CCS_exp_ and CCS_theo_ values showed a difference of 8%. As a matter of fact, the experimental CCSs determined with a traveling wave ion mobility setup, depend dramatically on the standard used to perform the mobility cell calibration. Polyalanine, a very widely-adopted CCS calibrant, was presently used and is structurally very different from that aromatic compound under investigation as much more unfolded. This might lead to underestimated CCSs, so that the actual experimental CCSs values might be higher than that reported in Table 1.^41^ Consequently, given the weak differences between the CCS values of the different structures, and the potential uncertainties associated with the estimate of both experimental and theoretical CCSs, the presence of the 1cK^+^ structure cannot be ruled out on the sole base of ion mobility measurements.

**Table 1:** Experimental (CCS_exp_) and theoretical (CCS_theo_) CCS values for the [1 + K]^+^ cation

CCS_exp_ (Å^2^)		CCS_theo_ (Å^2^)
199.5 ± 2.6	1aK^+^	220.2
	1bK^+^	207.0
	1cK^+^	216.7
	1dK^+^	200.1
	1eK^+^	199.1
	1fK^+^	198.7
	1gK^+^	206.9
	1hK^+^	212.1
	1iK^+^	211.8
	1jK^+^	211.1
	1kK^+^	215.8
	1lK^+^	207.6

In conclusion, taking into account the combined mass and IRMPD experiments, our results tend to confirmed that the binding of 1 with K^+^ most likely results in a combination of two main complexes involving a carbonyl–cation interaction (1cK^+^) and a mixed sulphur/carbonyl–cation interaction (1eK^+^).

### BLM experiments

Different methods have been reported to demonstrate the formation of ion channels (or nanopores) in model lipid membrane.^42,43^ Fluorescence spectroscopy, requiring lipid vesicles, and electrophysiology, requiring planar lipid bilayers, are the most used techniques. Fluorescence spectroscopy gives a mean analysis of interactions occurring between the systems studied and the lipid membrane. However, with unknown systems for nanochannel applications, performing planar lipid bilayer experiments (or ‘black lipid membrane’ BLM) presents the advantage to provide the view of unique events, allowing for the observation and characterization of unique nanochannels. If well-defined structures are obtained, then analysis of the molecular unit number forming the nanochannels can be determined, providing inputs on the channel features. First, tuning the concentration of the molecule is undertaken to find the adequate conditions to have unique events, witnessing the insertion of one channel in the lipid membrane.

We studied the interactions between 1 and the lipid membrane depending on the concentration of 1, ranging from 29 μM to 20 nM, until discrete and reproducible current signals were obtained. This choice was based on the concentration range found in the literature.^44–47^ It is worth mentioning that each experiment was performed at the same sampling frequency and the data analysis was performed at the same filter frequency. The molecules 1 and 3 were both solubilised in THF and added in the chambers of the BLM set-up, in order to overcome the potential detrimental effect of a change of solvents.

**Fig. 7:**
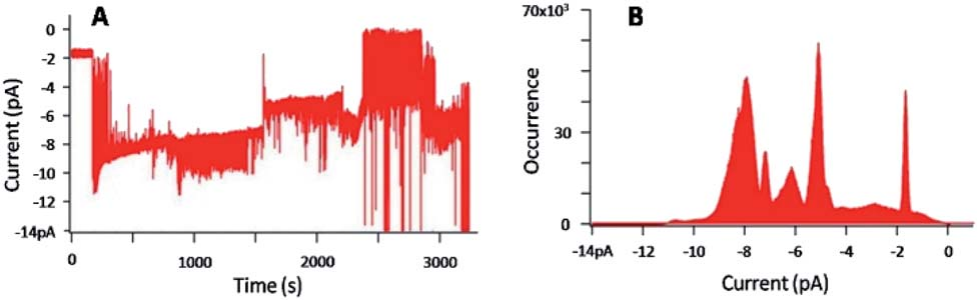
(A) Current–time trace for an applied voltage of −100 mV, in presence of 1 ([1] = 29 μM), with [KCl] = 1 M, (B) corresponding current distribution.

Fig. 7 gives a representative example of the current *versus* time trace and the corresponding current distribution, obtained with a concentration of 1 and KCl of 29 μM and 1 M, respectively, and an applied voltage of −100 mV.

Fig. 7A shows the perturbation of the membrane induced by the presence of 1. Defined and discrete current variations were detected, attributed to the insertion of 1 in the lipid bilayer and its pulling out of the membrane. The histogram (Fig. 7B) representing the number of events at each current, confirmed the discrete jumps and revealed different populations of current events. The typical analysis of the histogram consists in plotting the current as a function of the number of channels.^48^ However, despite the well-defined events, plotting this graph was here impossible due to too many populations of current events. The concentration of 1 was then gradually decreased until 20 nM to get access to possible defined permeation structures.

**Fig. 8:**
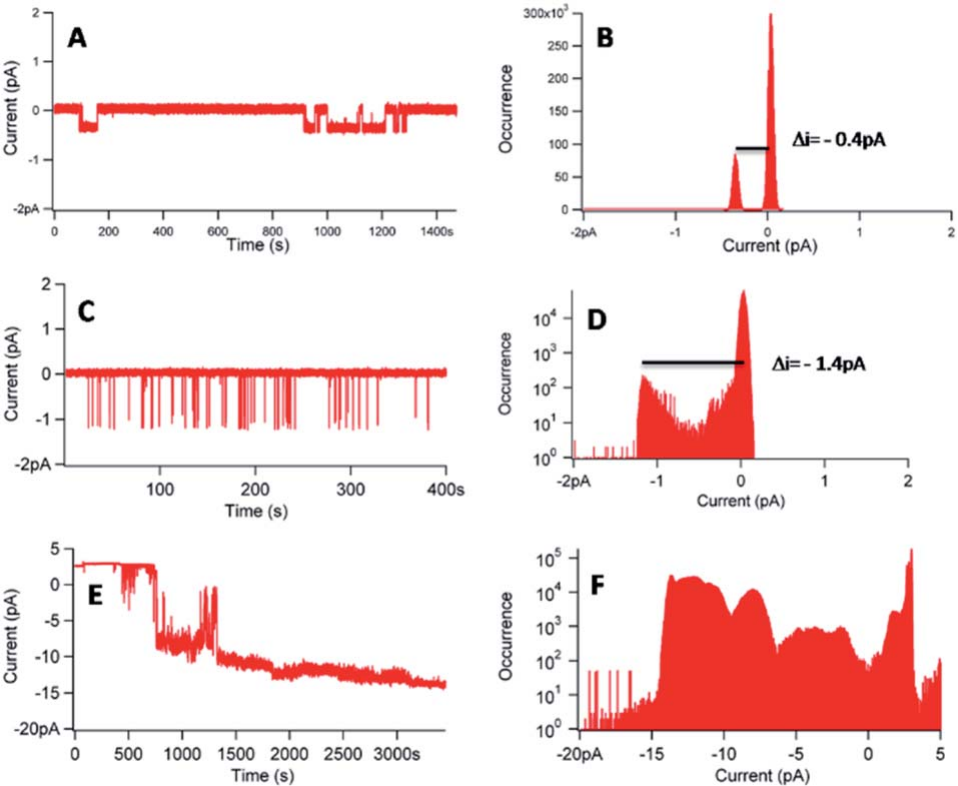
(A, C and E) Current–time traces for an applied voltage of −100 mV in presence of 1 ([1] = 20 nM–[KCl] = 1 M). (B, D and F) Corresponding current distributions.

Fig. 8 shows three different experiments, in the case of a concentration of 1 of 20 nM, with [KCl] = 1 M and an applied voltage of −100 mV.

The experiments, performed with a different membrane for each experiment, reveal different behaviours. In the three experiments, a flow of ions through the membrane in presence of 1 was observed (Fig. 8A, C and E), proving the interactions of 1 with the lipid bilayer. The corresponding histograms (Fig. 8B, D and F) gave different information. First, it was possible to obtain single channels at 20 nM of 1, considering the reproducible current jumps in each separate experiment revealed by the histograms. In Fig. 8B, the discrete current was equal to −0.4 pA for an applied voltage of −100 mV. In Fig. 8D, under identical conditions, a discrete current of −1.4 pA was obtained. However, we can decipher another population with a lower occurrence, around −0.4 pA, not detected during the data analysis. Noteworthy, these very low signals, at the pA level, require a low filter frequency of 300 Hz, to allow for the observation of the low current values. The difficulty to control or to reproduce the permeation structure was also proved with the trace of Fig. 8E and the corresponding histogram (Fig. 8F), which revealed different populations of current events, with a difficulty to access to a similar current jump, even at this concentration of 20 nM.

Another noticeable complementary point referred to the difference observed on the permeation lifetime, ranging from seconds (Fig. 8C) to minutes (Fig. 8A and E), comparable with other synthetic nanochannels.^49^ However, complete control of the permeation structure revealed difficult to set.

In conclusion, 1 was shown to be able to insert in a lipid membrane and to form single channels. However, the variabilities of the permeation structures and the permeation lifetimes have to be mentioned. The hydrophobic character of 1 can explain the difficulty to control the permeation structures, the self-assembly of 1 being possible, as shown with cyclic peptide architectures.^50,51^

In order to improve the control of the insertion, the Hydrophilic–Lipophilic Balance (HLB) was changed, by grafting PEG_550_ chains on 1, thus making 1 amphiphilic (3). It has been already demonstrated that the presence of PEG on a channel does not change the channel characteristics.^52,53^ In addition, the incorporation of PEG chains was also envisioned to induce a control of the permeation, as the PEG chains remain in contact with the aqueous solutions. A concentration of 20 nM was studied for the evaluation of 3 in a separate experiment, but in the same conditions as the ones used for 1 including the same membrane.

**Fig. 9:**
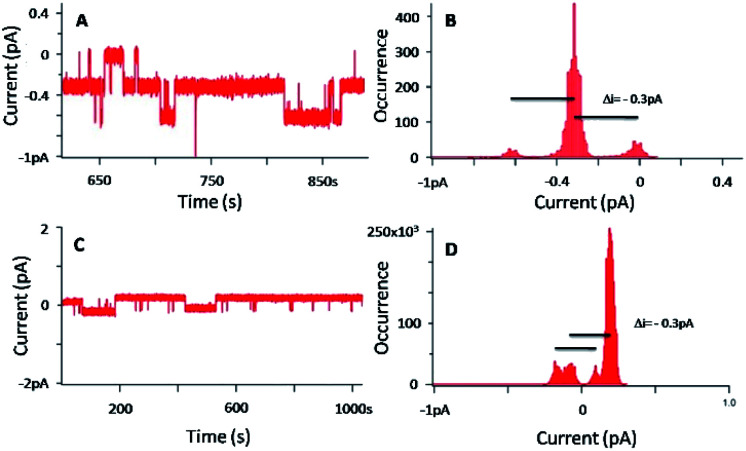
(A and C) Current–time traces for an applied voltage of −100 mV for two separate experiments, in presence of 3 ([3] = 20 nM–[KCl] = 1 M) (B and D) corresponding current distributions.

Fig. 9 shows the BLM results and the data analyses carried out in the case of a concentration of 3 of 20 nM, with [KCl] = 1 M and an applied voltage of −100 mV.

Fig. 9A and C show a flow of ions through the membrane induced by the presence of 3, and prove the ability of 3 to insert in the lipid bilayer as single nanochannels. The corresponding histograms (Fig. 9B and D) demonstrate that the discrete current is around −0.3 pA for an applied voltage of −100 mV. This current corresponds to the minimal current obtained in the case of 1. Indeed, considering the low current and the filter frequency used, currents of −0.3 pA and −0.4 pA can be assimilated. That tends to prove that the permeation structures of 3 correspond to the permeation structure giving the lowest signal for 1.

It is also necessary to mention that the BLM results in Fig. 9A were obtained with 3 added in both chambers, whereas the ones in Fig. 8C were obtained with 1 added in one chamber only. Despite different experimental conditions, the BLM data provide a rather similar trend, and the permeation structures appear to be the same. It is known that pore-mediated transmembrane lipid translocation (flip-flop) processes are involved in natural cells.^54,55^ The presence of defects in the membrane due the amphiphilic insertion of 3 in one leaflet of the bilayer allows for an increase flip-flop rate of the lipids, and 3 may thus follows this translocation trend, hence allowing the formation of the channel.

As reported by Y. El Ghoul *et al.*^44^ and by using the Hill's equation,^56^ we expected to get information about the number of molecules participating to the pore formation, by using different concentrations of pore-forming molecules. As reported as an example in ESI (Fig. S11†), the permeation structures vary dramatically in a short concentration range (histograms of the traces presented in Fig. S11B and D†), preventing from any mechanism analysis. However, the molecular structure of 3 is similar to the structure described in the work of Mayer and co-workers,^53,57^ nanopores that were best fitted by the Tabushi model.^58^

The presence of multiple permeation structures deserves a comment. Most molecules able to be inserted in lipid bilayers are reported to be amphiphilic. The carbon framework of 1 provides hydrophobicity only, driving to aggregates formation in aqueous solutions. A large set of structures may therefore destabilize the hydrophobic part of the lipid bilayer, allowing for transient pore formation. Grafting a hydrophilic polymer on helicene, compound 3, provides an amphiphilic compound, and its insertion in the lipid bilayer is then governed mostly by the unimers at the concentrations studied. Formation of aggregates cannot be excluded at higher concentration and a more complex pore structure would then be involved in nanochannel formation. Concomitant insertion by single molecules and aggregates may explain the large set of permeation structures recorded in the BLM study.

## Conclusions

In this paper, we succeeded in the preparation of helical pentacyclic architectures. The helical topology comprises two potential cation binding sites, namely a ketone and a carboxylic ester group. The latter was found essential to modulate the hydrophilic/hydrophobic balance of the overall molecular structure. Concerning the interaction of the potassium with the helical building block, the combined theoretical and IRMPD study shows that K^+^ expectedly exhibits a particularly strong affinity for the carbonyl groups and supports the formation of a mixture of structures of similar stabilities that are 1cK^+^ and 1eK^+^. Mass spectrometry experiments show that protonated multimers are not generated under electrospray conditions. On the other hand, formation of multimers with K^+^ is easily observed, but MS/MS experiments are not conclusive concerning their structure (losses of intact helicene units). Further, helical platforms 1 and 3 are able to interact with a lipid membrane and to form single channels. However, for these systems, despite an improvement obtained with the amphiphilic 3, variability in the permeation structures was evidenced. The fact that the systems are not based on a rigid macrocyclic scaffold could be an explanation for these results. Even if the proof of concept that using helicene-like architectures as synthetic nanochannels was obtained, it seems that the permeation structures cannot be predicted, making the deep characterization of the system difficult, in terms of structural parameters and number of molecules involved in the pore formation. However, the BLM analysis suggests that the minimal permeation structure obtained by 1 and 3 is the same.

## Conflicts of interest

There are no conflicts to declare.

## Supplementary Material

RA-010-D0RA05519K-s001

RA-010-D0RA05519K-s002
