# Supplementary material for: Helically shaped cation receptor: design, synthesis, characterisation and first application to ion transport
Source: RSC Adv. 2020 Aug 27;10(52):31670–9. doi: 10.1039/d0ra05519k (PMC9056421; doi:10.1039/d0ra05519k)
Supplement: RA-010-D0RA05519K-s001 [file RA-010-D0RA05519K-s001.pdf]

Electronic Supplementary Material (ESI) for RSC Advances.
This journal is © The Royal Society of Chemistry 2020

Helically shaped cation receptor: design, synthesis, characterisation and first application to ion transport

Hamza Boufroura,[a] Romain Plais,[a] Salomé Poyer,[b,c] Anne Gaucher,[a] Jérome Marrot,[a] Damien Prim,[a] Gilles Clavier,[d] François-Xavier Legrand,[e] Jean-Yves-Salpin,[b,c] Cécile Huin,[f,g] Philippe Guégan,[f]

[a] Université Paris-Saclay, UVSQ, CNRS, UMR 8180, Institut Lavoisier de Versailles, 78000, Versailles, France.
[b] Université Paris-Saclay, CNRS, Univ Evry, LAMBE, 91025, Evry-Courcouronnes, France
[c] Cergy Paris Université, CNRS, LAMBE, 95000 Cergy, France
[d] Université Paris-Saclay, CNRS, ENS Paris-Saclay, PPSM, 94235, Cachan, France
[e] Université Paris-Saclay, CNRS, Institut Galien Paris-Saclay, 92290, Châtenay-Malabry, France
[f] Sorbonne Université, CNRS, Institut Parisien de Chimie Moléculaire, Equipe Chimie des Polymères, 4 place Jussieu, F-75005 Paris, France
[g] Université Paris-Saclay, Université d’Evry Val d’Essonne, 91025, Evry, France

Electronic Supplementary Information

Table of contents

1. General Procedures, Materials and Instrumentation 3

1.1 General experimental procedures and materials 3

1.2 Instrumentation 3

1.3 Molecular modeling and software 5

2. Computational data 7

2.1 Computational data for 1 and 1xK^+ complexes 7

2.2 Determination of interaction energies 26

3. Photophysical analysis and procedures 27

3.1 General practical analysis procedure 27

3.2 Time dependant DFT analysis of 1 27

4. Mass spectrometry 30

5. IRMPD data 33

6. BLM experiments 35

7. Synthetic procedures and characterization data 36

7.1 Preparation of methyl 8-oxo-8H-indeno[2',1':7,8]naphtho[1,2-b]thiophene-2-carboxylate 1 36

7.2 Preparation of 8-Oxo-8H-indeno[2',1':7,8]naphtho[1,2-b]thiophene-2-carboxylic acid....... 37

7.3 Preparation of 2,5,8,11,14,17,20,23,26,29,32,35-Dodecaoxaheptatriacontan-37-yl 8-oxo-8H-indeno[2',1':7,8]naphtho[1,2-b]thiophene-2-carboxylate 3 38

7.4 ^{1}H NMR, ^{13}C NMR and mass spectra of 2,5,8,11,14,17,20,23,26,29,32,35-Dodecaoxa heptatriacontan-37-yl 8-oxo-8H-indeno[2',1':7,8]naphtho[1,2-b]thiophene-2-carboxylate 3 39

8. Bibliography 40

1. General Procedures, Materials and Instrumentation

1.1 General experimental procedures and materials

Unless otherwise noted, all starting materials were obtained from commercial suppliers and used without purification.

Reaction progress was carried out using pre-coated TLC sheets ALUGRAM® Xtra SIL G/UV$_{254}$ (0.20mm) from Macherey-Nagel® and visualized under 254 and 365 nm UV lamp from Fisher Bioblock Scientific®. Flash chromatography were proceeded using Silica 60M (0.04-0.063mm) for column chromatography silica gel from Macherey-Nagel®.

1.2 Instrumentation

^{1}H NMR spectra were recorded with Bruker AV-I 300MHz spectrometer at 298K, referenced to TMS signal and were calibrated using residual proton in MeOD (δ=3.31ppm), according to the literature. [1]

^{13}C NMR spectra were recorded with a Bruker AV-I 300MHz spectrometer at 75MHz and 298 K and were calibrated using MeOD (δ = 49.00 ppm). [1] ^{1}H NMR spectroscopic data are reported as follow: chemical shift δ [parts per million] (multiplicity, coupling constants in Hertz, integration). Multiplicities are reported as follow: s = singlet, d = doublet, t = triplet, q = quadruplet, quint = quintuplet, sext = sextuplet, hept = heptuplet, dd = doublet of doublet, td = triplet of doublet, tt = triplet of triplet, ddd = doublet of doublet of doublet, m = multiplet. ^{13}C NMR spectroscopic data are reported in terms of chemical shifts δ [ppm] and when it is necessary multiplicity and coupling constant in Hertz.

To check the structure of the product obtained during the synthesis, high resolution mass spectra (HRMS) were obtained with a Waters Xevo QTOF instrument fitted with an electrospray ionization source (ESI+), using Leucine Enkephaline solution as internal calibrant.

In order to study the interaction of **1** with the potassium cation, three different mass spectrometry were used.

ESI-QIT-MS(/MS) experiments.

ESI-QIT-MS(/MS) experiments were carried out using a quadrupole ion trap mass spectrometer (AmaZon Speed ETD, Bruker) equipped with an Apollo electrospray source. MS experiments were performed in enhanced resolution mode in the 50 to 2000 mass range in the positive ionization mode. Sample **1** was analyzed by direct infusion at 30 µM in $CH_3OH/CHCl_3$ 8:2 or at 150 µM in a methanolic solution of 1mM of KCl for both MS and MS/MS experiments. HDX experiments were performed by infusing **1** at 30 µM in $CH_3OD/CDCl_3$ 8:2. ESI source parameters were set as follows: capillary voltage 3.5 kV, end plate offset −100 V, nebulizer gas flow 5 psi, dry gas flow 5 L min^{-1} and dry gas temperature 150 °C. Data analysis v4.0 software was used to process data.

ESI-IMS-QTOF-MS experiments.

The hybrid IMS-MS instrument (Synapt G2-Si HDMS, Waters) was equipped with an ESI source operating in the 50-2000 mass range in the positive ionization mode. Source conditions were the following: capillary voltage 3.0 kV, sampling cone 30 V and source temperature 50°C. Sample **1** at 150 µM in a methanolic solution of 1mM of KCl was analyzed by direct infusion. Six different IMS conditions were used for CCS measurement, similar for the analysis of **1** and the poly-DL-alanine calibrant (100 µg mL^{-1} in MeOH); wave velocity was varied from 600 to 1200 m s^{-1}, wave height was set at 40 V, and gas flow was varied from 70 to 90 mL min^{-1}. The N_2 modified version of the Mobcal software was used to convert Cartesian coordinates in CCS values from optimized DFT structures using calculated Mulliken partial charges.

ESI-QqQ-MS(/MS) experiments.

ESI-QqQ-MS(/MS) experiments were carried out using an API-2000 triple quadrupole instrument fitted with a turbo ionspray source. Sample **1** at 150 µM in a methanolic solution of 1mM of KCl was analyzed by direct infusion. The ESI conditions were as follows: flow rate: 300 □l/h; sprayer probe voltage: 5.0 kV; pressure of GAS1 (nebulizing gas, air): 1,4 bar; pressure of GAS2 (air): 0.7 bar, temperature of GAS2: 50°C; pressure of curtain gas (N_2): 1,4 bar. In order to record low-energy CID spectra, precursor ions were selected by the first mass filter (Q1), then were allowed to collide with nitrogen in the collision cell (Q2), at different collision energies. Finally, the fragment ions were analyzed by the second mass filter (Q3). MS/MS spectra were carried out by introducing nitrogen as collision gas in the second quadrupole. The collision energy was varied from 10 to 20 eV (laboratory frame) depending on the precursor ion.

IRMPD experiments

The electron energy of the CLIO FEL was set at 44 MeV to optimize the laser power in the frequency region of interest (800-1900 cm^{-1}). The FEL beamline was coupled to an experimental platform based on a modified Bruker Esquire 3000+ quadrupole ion trap. A 2 mm tapered hole is drilled in the annular electrode of the quadrupole ion trap to allow access of the IR laser to the center of the trap. Multistage mass spectrometry was carried out using the standard Bruker Esquire Control (v5.2) software, and mass-selected ions were irradiated (typical irradiation time 250 ms) using the MS2 step, where the excitation amplitude was set to 0 to avoid any CID-like process. 2 attenuators were used to decrease the irradiation power, as the loss of K^+ is a facile dissociation process. Mass spectra were recorded after 10 accumulations, using the standard mass range (m/z 50-3000) and the normal scan resolution (13000 Th/s), the accumulation time being typically of 20 ms. This sequence was repeated ten times for each photon energy.

IRMPD spectra are obtained by plotting the photofragmentation yield R

$$R = -\ln[I_{precursor}/(I_{precursor} + \Sigma I_{fragments})]$$

(where Iprecursor and Ifragments are the integrated intensities of the mass peaks of the precursor and of the fragment ions, respectively) as a function of the frequency of the IR radiation. Spectra were externally calibrated in energy using polystyrene as reference in the 800 to 1700 cm^{-1} range. Finally, for a better comparison with the experimental IRMPD signal, computed spectra with the B3LYP functional were convoluted with a 15 cm^{-1} Lorentzian function.

BLM experiments

The interactions of helicene derivatives with black lipid membranes were carried out using a home-made set-up. Two chambers containing 1 mL of a 1M KCl solution are connected by a 150 µm wide hole, drilled in a Delrin wall, on which a film of a 1% solution of diphytanoylphosphatidylcholine lipid in decane is painted. The decane film is thinned in order to get a planar bilayer. The bilayer formation is controlled by measuring the current between two Ag-AgCl electrodes and the bilayer integrity is witnessed by a strong current variation from the saturation value of the amplifier to about 0 pA. The membrane thickness is estimated before each experiment by the measurement of the capacity of the membrane (around 5 nm). Helicene derivatives from a stock solution were added in both chambers, at controlled concentrations. Then, a voltage was applied to the lipid bilayer to assess the formation of channels through the membrane, and the current intensity versus time was recorded.

Data acquisition: The ionic current through the membrane was measured with a BLM 120 amplifier (Biologic). Data were acquired at 1,500 Hz and filtered at 300 Hz with the Measurement Computing Digitizer. The formation of channels is witnessed by discrete current intensity recording under applied voltage.

Photophysical experiments

UV-Visible spectra were recorded at 22°C on a Cary 4000 (Agilent) double-beam spectrometer using a 10 mm path quartz cell. The titration experiment was done by addition of aliquots of a potassium tetrakis(4-biphenylyl)borate (KTBPB) THF (spectroscopic grade) solution (c=1.63 mM) in a THF solution (c=3.6µM) of compound **1**.

1.3 Molecular modeling and software

All calculations were carried out using Gaussian 09® program:

Gaussian 09, Revision D.01, M. J. Frisch, G. W. Trucks, H. B. Schlegel, G. E. Scuseria, M. A. Robb, J. R. Cheeseman, G. Scalmani, V. Barone, B. Mennucci, G. A. Petersson, H. Nakatsuji, M. Caricato, X. Li, H. P. Hratchian, A. F. Izmaylov, J. Bloino, G. Zheng, J. L. Sonnenberg, M. Hada, M. Ehara, K. Toyota, R. Fukuda, J. Hasegawa, M. Ishida, T. Nakajima, Y. Honda, O. Kitao, H. Nakai, T. Vreven, J. A. Montgomery, Jr., J. E. Peralta, F. Ogliaro, M. Bearpark, J. J. Heyd, E. Brothers, K. N. Kudin, V. N. Staroverov, T. Keith, R. Kobayashi, J. Normand, K. Raghavachari, A. Rendell, J. C. Burant, S. S. Iyengar, J. Tomasi, M. Cossi, N. Rega, J. M. Millam, M. Klene, J. E. Knox, J. B. Cross, V. Bakken, C. Adamo, J. Jaramillo, R. Gomperts, R.

E. Stratmann, O. Yazyev, A. J. Austin, R. Cammi, C. Pomelli, J. W. Ochterski, R. L. Martin, K. Morokuma, V. G. Zakrzewski, G. A. Voth, P. Salvador, J. J. Dannenberg, S. Dapprich, A. D. Daniels, O. Farkas, J. B. Foresman, J. V. Ortiz, J. Cioslowski, and D. J. Fox, Gaussian, Inc., Wallingford CT, 2013.

To evaluate the geometry of the complex, optimizations were calculated at M06-2X/6-311G++(d,p) level of theory using Gaussian® software without any solvent correction. In order to find most propitious binding sites, optimizations were carried out at M06-2X/6-31G+(d,p) level of theory. Stationary points were verified by a harmonic vibrational frequencies calculation. None of the predicated geometry has any imaginary frequency implying that the optimized geometry of each of the molecules and complexes under study lay at a minimum local point on the potential energy surface.

Electrostatic Potentials Surfaces (ESP) were thus calculated using Gaussview® software from optimized structures using a fine grid for Total Density and a medium grid of ESP. NCIplots were generated from total densities using the script developed by Rzepa available on the website application of the Imperial College London.[2,3]

In order to calculate UV-Visible spectrum of **1**, optimization and frequencies calculations of molecule **1** were carried out using DFT calculation at M06-2X/6-311++g(d,p) level of theory and a IEFPCM solvent model for THF using Gaussian software. Theoretical UV-Visible spectrum was subsequently calculated using time dependant DFT calculation at PBE1PBE/6-311++g(d,p) level of calculation with a IEFPCM model for THF and solving was done on the 24 first singlet states. Electronic transitions were then extracted using GAUSSSUM software [4] and were attributed after calculation of corresponding molecular orbitals using Gaussview software.

2. Computational data

2.1 Computational data for <u>1</u> and <u>1xK⁺</u> complexes

Figure S1. Optimized geometries and relative energy values (at the M06-2X/6-311++G(2df,2pd)//M06-2X/6-31+G(d,p) level) at 298 K (kJ mol^{-1}) of the various structures of the [**1**+K]$^+$ complex.

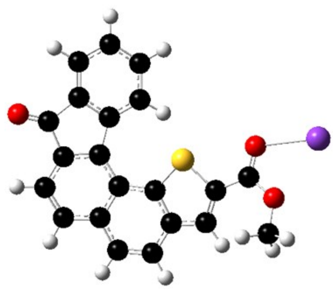

1aK$^+$ (+39.6)

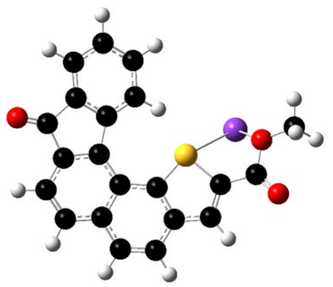

1bK$^+$ (+57.7)

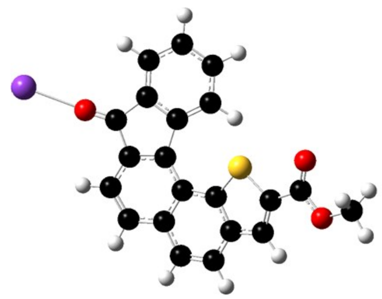

1cK$^+$ (+0.0)

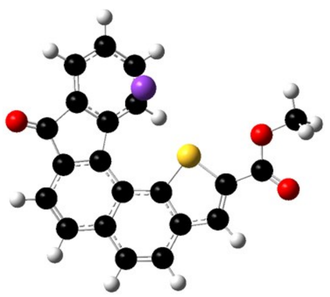

1dK$^+$ (+27.7)

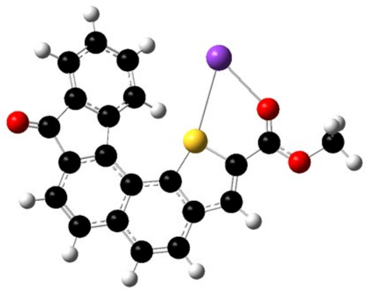

1eK$^+$ (0.0)

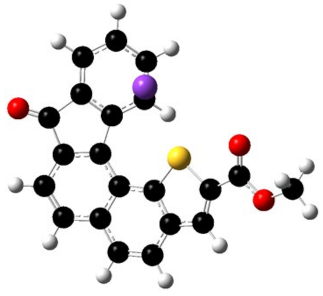

1fK$^+$ (+16.5)

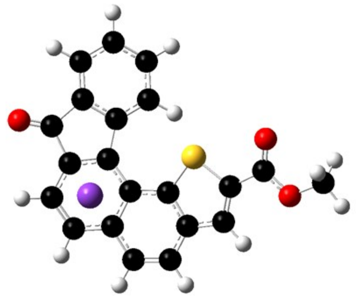

1gK$^+$ (K$^+$ above) (+30.6)

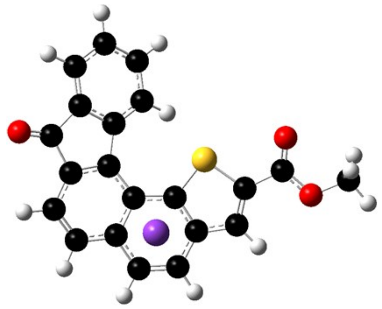

1hK$^+$ (K$^+$ above) (+33.4)

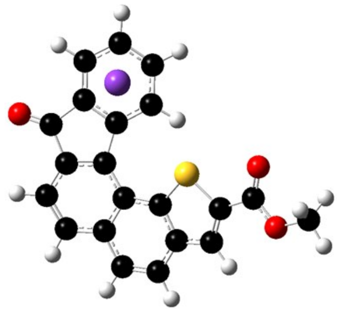

1iK$^+$ (K$^+$ under) (+34.4)

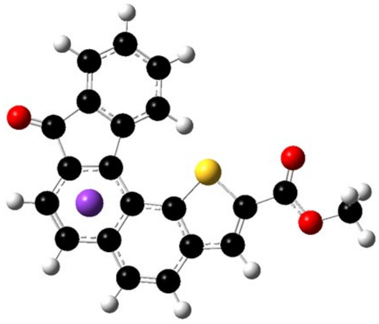

1jK$^+$ (K$^+$ under) (+35.0)

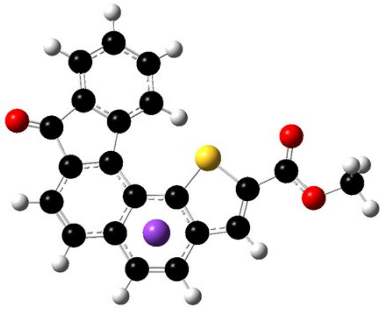

1kK$^+$ (K$^+$ under) (+40.2)

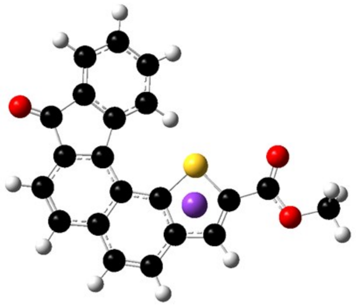

1lK$^+$ (K$^+$ under) (+47.0)

Table S1: Computational data associated with the various structures optimized for the [**1**+K]$^+$ complex. Data obtained at the M06-2X/6-311++G(2df,2pd)//M06-2X/6-31+G(d,p) level of calculation.

Structure New	E+ZPE	ΔE+ΔZPE	H°$_{298}$	ΔH°$_{298}$	G°$_{298}$	ΔG°$_{298}$

**1aK⁺**	-2030.778331	40.0	-2030.7560818	39.6	-2030.8311018	40.8
**1bK⁺**	-2030.771631	58.9	-2030.7491760	57.8	-2030.8247030	57.6
**1cK⁺**	-2030.793553	0.1	-2030.7711599	0.0	-2030.8460319	1.6
**1dK⁺**	-2030.783057	28.2	-2030.7606122	27.7	-2030.8350712	30.4
**1eK⁺**	-2030.793684	**0.0**	-2030.7711733	**0.0**	-2030.8466523	**0.0**
**1fK⁺**	-2030.787350	15.5	-2030.7648913	16.5	-2030.8401163	17.2
**1gK⁺**	-2030.781987	30.7	-2030.7595234	30.6	-2030.8343934	32.2
**1hK⁺**	-2030.780994	33.7	-2030.7584597	33.4	-2030.8339877	33.3
**1iK⁺**	-2030.780597	35.4	-2030.7580548	34.4	-2030.8334298	34.7
**1jK⁺**	-2030.780362	36.2	-2030.7578332	35.0	-2030.8329902	35.9
**1kK⁺**	-2030.778361	41.0	-2030.7558690	40.2	-2030.8309290	41.3
**1lK⁺**	-2030.775887	47.9	-2030.7532823	47.0	-2030.8292623	45.7

Electronic energies reported afterwards are defined as the sum of electronic and zero-point energy calculated from Gaussian software. Quadrupole moment was extracted from Gaussian calculation using a field-independent basis and reporting ZZ value.

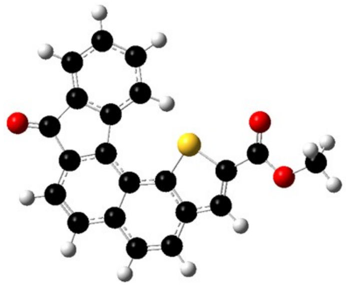

1-B3LYP

B3LYP/6-311++G(d,p)
Charge: 0
Spin Multiplicity : Singlet
Imaginary frequencies: 0
Electronic Energy: -1431.638515 Hartree
Quadrupole moment: -155.3317 Buckingham

Symbol	*X*	*Y*	*Z*
C	-3.06497300	0.97285500	-0.27101800
C	-3.29620800	2.34691000	-0.32658300
C	-2.24316200	3.18749100	-0.04885100
C	-0.94155300	2.67893600	0.17084600
C	-0.68169200	1.25523000	0.13026900
C	-1.82172100	0.40356800	0.02544400
C	0.11886700	3.60862200	0.40791800
C	1.41401100	3.20651900	0.51595700
C	1.73116100	1.83295800	0.33427800
C	0.70796800	0.88135000	0.14385600
C	3.03901100	1.27644100	0.23623700
C	3.02869500	-0.05307500	-0.06770700
S	1.40672100	-0.68111300	-0.23005800
C	-2.04410500	-1.07330500	0.19999600
C	-3.37709900	-1.36871300	-0.16074000
C	-4.08373500	-0.09851500	-0.47892600
C	-1.27264900	-2.09482300	0.74321500
C	-1.80747800	-3.38893200	0.83104300
C	-3.10160100	-3.67401900	0.40665000
C	-3.91097200	-2.64155700	-0.07906300
O	-5.24029500	0.05042900	-0.81485700
C	4.15968700	-0.97244400	-0.26530100
O	4.04768600	-2.14922300	-0.52764100
H	-4.28992700	2.71786400	-0.54823700
H	-2.38623900	4.26191700	-0.02013300
H	-0.13716500	4.65944400	0.48255500
H	2.21067000	3.92095200	0.68933800
H	3.95019600	1.84383000	0.36689300
H	-0.27635600	-1.92169500	1.11892200
H	-1.19345000	-4.17981200	1.24715400

H	-3.48758500	-4.68401900	0.47943900
H	-4.94160500	-2.81461000	-0.36765700
C	6.51446000	-1.16740900	-0.29411200
H	6.53432900	-1.59021200	-1.29931600
H	7.36083200	-0.50184500	-0.14069600
H	6.52367700	-1.97690900	0.43682500
O	5.34555900	-0.34440800	-0.11807700

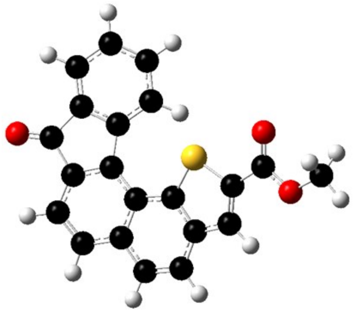

1

M062X/6-311++G(d,p)
Charge: 0
Spin Multiplicity : Singlet
Imaginary frequencies: 0
Electronic Energy corrected by ZPE : -1430.930764 Hartree
Quadrupole moment: -155.1030 Buckingham

Symbol	*X*	*Y*	*Z*
C	-3.04452900	0.96358300	-0.28532100
C	-3.28756600	2.33422900	-0.35201500
C	-2.24694900	3.17964900	-0.05694000
C	-0.94741500	2.67685800	0.18193200
C	-0.68440700	1.26692900	0.15147700
C	-1.81133600	0.40966300	0.03683800
C	0.11070600	3.61093100	0.42307100
C	1.40083500	3.20977200	0.53842200
C	1.71786200	1.83448800	0.35808200
C	0.70247100	0.88821700	0.16993600
C	3.02422500	1.27279800	0.24617100
C	2.99028500	-0.04789900	-0.06839900
S	1.37867600	-0.66346500	-0.22317600
C	-2.01522800	-1.06829900	0.22067200
C	-3.32461400	-1.38595900	-0.17990500
C	-4.04319000	-0.12412900	-0.51892700
C	-1.25219900	-2.06199700	0.81622700
C	-1.77185300	-3.35973900	0.89889600
C	-3.04006900	-3.67027500	0.42599900
C	-3.84682300	-2.65923800	-0.10056000
O	-5.18390100	0.00563700	-0.88056500
C	4.12090500	-0.96971300	-0.28519500

O	4.00282600	-2.13428300	-0.56240400
H	-4.28005500	2.69425200	-0.59600500
H	-2.39384500	4.25346500	-0.03454100
H	-0.14801900	4.66119900	0.49311900
H	2.19840100	3.92269800	0.71175300
H	3.94473900	1.82661700	0.37190500
H	-0.28200200	-1.86253800	1.24475200
H	-1.16729000	-4.13488900	1.35510900
H	-3.41287000	-4.68443900	0.50004900
H	-4.86603500	-2.84628600	-0.41939100
C	6.44859000	-1.17761200	-0.33455700
H	6.46039300	-1.56747800	-1.35258000
H	7.30605000	-0.53379700	-0.15906200
H	6.44060400	-2.01098200	0.36804600
O	5.29893700	-0.35084700	-0.13550300

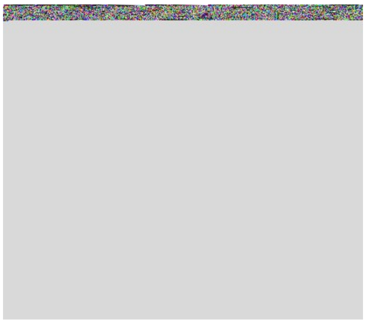

1

M062X/6-31+G(d,p)
Charge: 0
Spin Multiplicity : Singlet
Imaginary frequencies: 0
Electronic Energy corrected by ZPE : -1430.676209 Hartree
Quadrupole moment: -154.9766 Buckingham

Symbol	*X*	*Y*	*Z*
C	-3.04999900	0.96558300	-0.27482200
C	-3.29565600	2.33762900	-0.34039100
C	-2.25025300	3.18424500	-0.05373200
C	-0.94774000	2.68042900	0.17761600
C	-0.68288000	1.26770100	0.14958100
C	-1.81225700	0.40845000	0.03809500
C	0.11063300	3.61887200	0.40946600
C	1.40522900	3.22076800	0.51994700
C	1.72298200	1.84329700	0.34592200
C	0.70677500	0.89183900	0.16603300
C	3.03204800	1.28235700	0.23758500
C	2.99845200	-0.04457200	-0.06402800
S	1.38690700	-0.66446800	-0.20829600

C	-2.01823800	-1.07199400	0.21552800
C	-3.33470700	-1.38517300	-0.17509600
C	-4.05132200	-0.12008900	-0.50269800
C	-1.25073100	-2.07675900	0.79211100
C	-1.77380000	-3.37636000	0.86809300
C	-3.05037600	-3.68014200	0.40588000
C	-3.86088500	-2.65996800	-0.10372600
O	-5.20156200	0.01427500	-0.85616000
C	4.12875500	-0.96919900	-0.27208400
O	4.00687900	-2.14565200	-0.52692600
H	-4.29202800	2.69742100	-0.57730800
H	-2.39627700	4.26025700	-0.03234700
H	-0.15079600	4.67075600	0.47614200
H	2.20346500	3.93753000	0.68605400
H	3.95321600	1.84058200	0.35653500
H	-0.27240100	-1.88599100	1.21074600
H	-1.16383800	-4.15826000	1.30997200
H	-3.42571100	-4.69585000	0.47404500
H	-4.88535700	-2.84099700	-0.41557500
C	6.45534700	-1.18525800	-0.33681800
H	6.44521300	-1.61306100	-1.34138400
H	7.31778500	-0.53544300	-0.20151700
H	6.45905500	-1.99359100	0.39730700
O	5.31080000	-0.34884800	-0.14500200

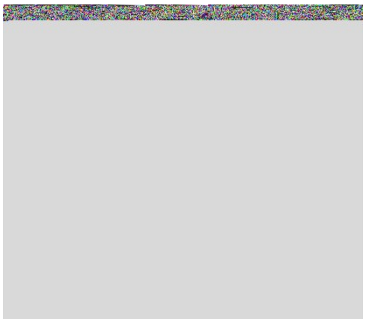

M062X/6-311++G(d,p) scrf=(iefpcm,solvent=thf)
Charge: 0
Spin Multiplicity : Singlet
Imaginary frequencies: 0
Electronic Energy corrected by ZPE : -1431.215812 Hartree

Symbol	*X*	*Y*	*Z*
C	-3.04570600	0.96386300	-0.28674900
C	-3.28801700	2.33552700	-0.35317300
C	-2.24732400	3.18116700	-0.05631800
C	-0.94786400	2.67751100	0.18339400
C	-0.68555000	1.26667600	0.15326200
C	-1.81212100	0.41032400	0.03821900
C	0.11028600	3.61225500	0.42460900
C	1.40065700	3.21005400	0.54026400
C	1.71622500	1.83376200	0.35988200

C	0.70116300	0.88729000	0.17252600
C	3.02383700	1.27367900	0.24737600
C	2.99080600	-0.04721900	-0.06743900
S	1.37821200	-0.66462900	-0.21907200
C	-2.01503000	-1.06738000	0.22347300
C	-3.32303800	-1.38680300	-0.18289000
C	-4.03784800	-0.12559100	-0.52435600
C	-1.25410100	-2.05787000	0.82600600
C	-1.77309800	-3.35719000	0.90945700
C	-3.03833600	-3.67067000	0.43000000
C	-3.84421600	-2.66119500	-0.10325600
O	-5.18006700	0.00150900	-0.89522500
C	4.12153400	-0.96756100	-0.28882000
O	3.99541800	-2.13329100	-0.57625800
H	-4.27770800	2.70295900	-0.59714100
H	-2.39490400	4.25468600	-0.03350300
H	-0.14762600	4.66267800	0.49325100
H	2.19924100	3.92167200	0.71305700
H	3.94062900	1.83382900	0.37342100
H	-0.28733000	-1.85628100	1.26153200
H	-1.17104500	-4.13010500	1.37256200
H	-3.41102700	-4.68468700	0.50458100
H	-4.86085700	-2.85671000	-0.42511900
C	6.45484400	-1.17771400	-0.33776300
H	6.46857000	-1.55932000	-1.35825300
H	7.30618600	-0.52748700	-0.16008900
H	6.45290400	-2.00994100	0.36536400
O	5.29576700	-0.35756000	-0.13485800

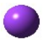

Potassium cation

M062X/6-31+G(d,p)

Charge : +1

Spin multiplicity : Singlet

Imaginary frequencies : 0

Electronic energy corrected by ZPE : -599.701164 Hartree

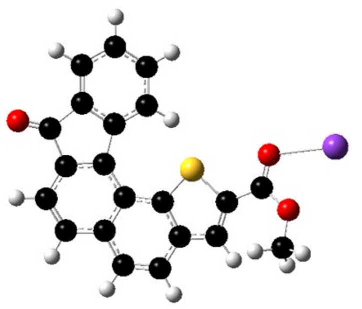

1aK$^+$

M062X/6-31+G(d,p)

Symbol	X	Y	Z
C	-3.05956000	0.97314900	-0.25848000
C	-3.31561700	2.34204100	-0.32512400
C	-2.27695900	3.20147300	-0.04335900
C	-0.97086700	2.70865900	0.17705400
C	-0.69976400	1.29748500	0.15683900
C	-1.81847900	0.42541000	0.05612900
C	0.08537300	3.65856000	0.39026300
C	1.38455900	3.27748000	0.47280200
C	1.70606500	1.89881000	0.28948100
C	0.69272000	0.93415000	0.14999400
C	3.00855700	1.35707000	0.13128800
C	2.99021000	0.01489200	-0.16166200
S	1.36983200	-0.62006700	-0.20700900
C	-2.00931800	-1.05473900	0.23577200
C	-3.31960100	-1.38256300	-0.16277300
C	-4.05021000	-0.12566600	-0.49322700
C	-1.23547100	-2.04734700	0.82548000
C	-1.74575300	-3.35144200	0.90612900
C	-3.01504100	-3.67065200	0.43351100
C	-3.83284000	-2.66260600	-0.08815800
O	-5.19619800	0.00019600	-0.85396800
C	4.05580300	-0.96707000	-0.32369800
O	3.86313800	-2.16876000	-0.19469300
H	-4.31551000	2.69334900	-0.56049200
H	-2.43494500	4.27536700	-0.02212600
H	-0.18596600	4.70714300	0.46416300
H	2.17856000	4.00200700	0.62235500
H	3.89858800	1.96154000	0.24738800
H	-0.26822000	-1.84115200	1.26400900
H	-1.13741600	-4.12402000	1.36615700
H	-3.38235900	-4.68864100	0.50806600
H	-4.85374200	-2.85682800	-0.40332400
C	5.69951800	0.69306400	-1.10448100
H	4.90847200	1.11366200	-1.72628500
H	5.92532300	1.34717700	-0.26104800
H	6.59980200	0.54361300	-1.69856300
O	5.32800500	-0.61232000	-0.62765400
K	6.11653700	-3.37177800	-0.40648700

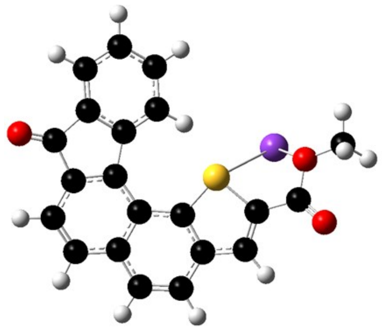

1bK$^+$

M062X/6-31+G(d,p)

Symbol	X	Y	Z
C	-3.00578000	0.93900500	-0.35727900
C	-3.28375200	2.29323500	-0.54625900
C	-2.27394000	3.19113300	-0.29240900
C	-0.96989200	2.74416100	0.02735400
C	-0.67207200	1.34050200	0.14142900
C	-1.77288100	0.43949500	0.05288900
C	0.05487700	3.73231000	0.19204500
C	1.35930200	3.39179200	0.35880000
C	1.71315100	2.01280000	0.32750300
C	0.72587900	1.01952500	0.24263200
C	3.04360900	1.49210500	0.28431000
C	3.07359500	0.13810300	0.14484700
S	1.47174400	-0.55465300	0.03896100
C	-1.95441700	-1.02904500	0.32901000
C	-3.24366000	-1.40593900	-0.09317600
C	-3.97417200	-0.18692100	-0.54585900
C	-1.19648000	-1.97750800	1.00504400
C	-1.69098400	-3.28111100	1.15644100
C	-2.93947200	-3.64704100	0.66472300
C	-3.74565100	-2.68443200	0.04802900
O	-5.10665800	-0.10681000	-0.95522900
C	4.30044100	-0.67339900	0.05140700
O	5.38145000	-0.26437300	-0.27155600
H	-4.27761900	2.60247900	-0.85419600
H	-2.44760500	4.25984000	-0.37163500
H	-0.23844500	4.77667200	0.14999400
H	2.13453200	4.14422300	0.45893000
H	3.94294500	2.09768900	0.32812500
H	-0.24259700	-1.72369000	1.44693900
H	-1.09133300	-4.02010400	1.67982100
H	-3.29769200	-4.66294700	0.79082300
H	-4.75100900	-2.91181000	-0.29367600
C	5.17710800	-2.86730700	0.17095200
H	6.05176900	-2.57036100	0.75116000
H	4.82651200	-3.85658000	0.45890200
H	5.42215300	-2.84370300	-0.89112500
O	4.07568800	-1.96803700	0.43215000
K	2.69156500	-1.66715600	2.79386300

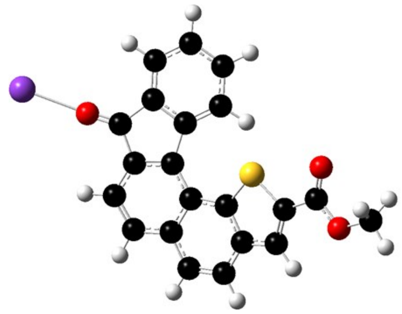

1cK^{+}
M062X/6-31+G(d,p)

	Symbol *X*	*Y*	*Z*
C	2.34407000	0.90284300	-0.06211700
C	2.62080800	2.27289000	-0.03562800
C	1.57322900	3.13688400	-0.25019000
C	0.24605000	2.65916900	-0.37337400
C	-0.04665200	1.25023200	-0.30217900
C	1.06736600	0.37309800	-0.27066400
C	-0.80652300	3.61366200	-0.54416400
C	-2.11270700	3.23428300	-0.55778900
C	-2.44578800	1.86709400	-0.33999100
C	-1.43907200	0.89776200	-0.21491800
C	-3.75638800	1.33380200	-0.13479000
C	-3.72419200	0.01248700	0.18513700
S	-2.11912100	-0.63576400	0.23264000
C	1.22482000	-1.11454200	-0.44565100
C	2.56177700	-1.45109500	-0.14659600
C	3.30742200	-0.20094200	0.10729100
C	0.39927700	-2.10430700	-0.95419200
C	0.88909300	-3.41852300	-1.05119800
C	2.18662600	-3.74798900	-0.67769600
C	3.05304500	-2.73923600	-0.24083400
O	4.50173500	-0.09696000	0.39253400
C	-4.84767000	-0.90456500	0.48134000
O	-4.69972000	-2.07770800	0.73457900
H	3.63522100	2.62949200	0.11085200
H	1.73965300	4.20860000	-0.29870300
H	-0.53511300	4.65963500	-0.64665100
H	-2.90657200	3.96410600	-0.68194100
H	-4.67370600	1.90677000	-0.20119000
H	-0.60367000	-1.89651700	-1.29988600
H	0.23133800	-4.19079600	-1.43692800
H	2.53311100	-4.77244100	-0.75748800
H	4.09081300	-2.95393900	-0.00300300
C	-7.16634500	-1.10621400	0.70078700
H	-7.08396100	-1.54460800	1.69675800
H	-8.02676600	-0.44407800	0.63681700
H	-7.23175500	-1.90326900	-0.04204500
O	-6.02492900	-0.27881300	0.43311500
K	6.85218600	-0.05089900	1.13066600

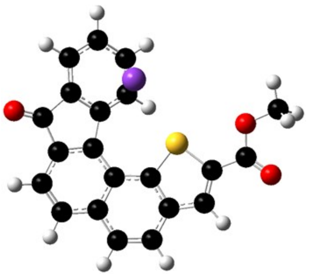

1dK^{+}

M062X/6-31+G(d,p)

Symbol	X	Y	Z
C	-2.94964500	1.01870000	-0.22496300
C	-3.27769600	2.37407100	-0.11976400
C	-2.29463900	3.23379200	0.31875700
C	-0.96215900	2.79076100	0.51685900
C	-0.61880000	1.41060200	0.32887300
C	-1.69061000	0.51410200	0.08730700
C	0.05245400	3.74632100	0.84517300
C	1.37013900	3.40411100	0.87559100
C	1.75377000	2.07651800	0.52838700
C	0.77980900	1.10460000	0.28331200
C	3.08709400	1.60413500	0.28692100
C	3.11670100	0.33158100	-0.18188800
S	1.52470600	-0.36909900	-0.29096700
C	-1.77785800	-0.98001400	0.07054200
C	-3.00779800	-1.34439900	-0.51514700
C	-3.82309500	-0.09299800	-0.70920400
C	-0.98105900	-1.98236500	0.62244200
C	-1.36364300	-3.32441500	0.45119400
C	-2.53190700	-3.66981800	-0.23070200
C	-3.39426200	-2.66109500	-0.69059500
O	-4.93707000	-0.02019700	-1.16507300
C	4.33740400	-0.42418600	-0.53907000
O	5.45138000	0.01699100	-0.42164100
H	-4.28194600	2.71275800	-0.35310000
H	-2.51312200	4.28653300	0.47079400
H	-0.25308700	4.76961300	1.03991200
H	2.13569700	4.13919900	1.10200400
H	3.98922900	2.18802700	0.43380800
H	-0.09654700	-1.75113500	1.20291900
H	-0.74870400	-4.10583600	0.88776000
H	-2.80731600	-4.71397600	-0.33818300
H	-4.35921900	-2.89137300	-1.13415700
C	5.19555500	-2.44481600	-1.35715100
H	5.82716700	-2.58552600	-0.47871400
H	4.79981100	-3.39646200	-1.70530000
H	5.77027100	-1.95056200	-2.14191800
O	4.04855800	-1.64835700	-1.01335200
K	-0.49412200	-2.12566700	-2.41894900

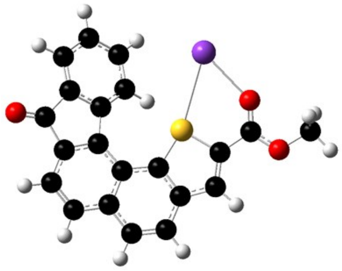

1eK+

M062X/6-31+G(d,p)

Symbol	*X*	*Y*	*Z*
C	-3.06136600	1.01905000	-0.28184800
C	-3.31637400	2.39194000	-0.34600300
C	-2.29296400	3.25113400	-0.01357200
C	-0.98992600	2.76292000	0.25191700
C	-0.72399000	1.35366500	0.23733100
C	-1.83412800	0.48096100	0.08786200
C	0.07207100	3.69828500	0.48218700
C	1.37029100	3.30279400	0.58337500
C	1.68805700	1.92357000	0.41129200
C	0.65853300	0.98503800	0.25927500
C	2.99463300	1.35091600	0.27184000
C	2.94613100	0.02314600	-0.02606900
S	1.32094300	-0.57755900	-0.11044500
C	-2.00995000	-1.00146200	0.24430900
C	-3.28072500	-1.34887000	-0.24828600
C	-4.01549300	-0.09091800	-0.59930500
C	-1.26909100	-1.97988200	0.90336400
C	-1.76127400	-3.29461500	0.94329300
C	-2.97968400	-3.63660800	0.35919900
C	-3.77305200	-2.63951500	-0.22028500
O	-5.13796600	0.01048600	-1.02983700
C	4.01202400	-0.94702900	-0.28819600
O	3.77322300	-2.11559200	-0.59349700
H	-4.30364200	2.74677100	-0.62440800
H	-2.45591100	4.32439200	0.00471700
H	-0.18478500	4.75116600	0.54615600
H	2.16706700	4.02266200	0.73892000
H	3.92003100	1.90841900	0.36413400
H	-0.36584300	-1.73542100	1.44935700
H	-1.20633800	-4.05086000	1.49332300
H	-3.34051900	-4.65839000	0.41344700
H	-4.76617200	-2.85021400	-0.60613100
C	6.32116100	-1.35037600	-0.41992400
H	6.26619300	-1.72740600	-1.44209100
H	7.21807200	-0.75396800	-0.27378000
H	6.28539400	-2.17971900	0.28779700
O	5.22461300	-0.45134100	-0.17191600
K	1.69870100	-3.59653000	-1.09757500

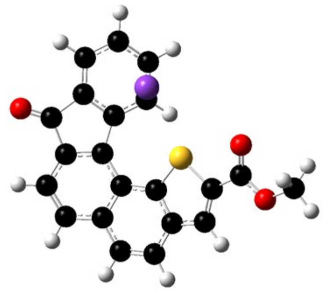

1fK⁺

M062X/6-31+G(d,p)

Symbol	*X*	*Y*	*Z*
C	-3.06802500	0.99966600	-0.26980500
C	-3.31919500	2.37302500	-0.36718100
C	-2.30550900	3.23584500	-0.01151600
C	-1.00705100	2.75597700	0.29815200
C	-0.74439500	1.34825200	0.32299600
C	-1.85372900	0.48049200	0.16519300
C	0.06096800	3.68723000	0.50954700
C	1.35793700	3.27995700	0.60720400
C	1.66681600	1.89604400	0.45941100
C	0.63208000	0.96247400	0.35235800
C	2.96737700	1.30742000	0.28171000
C	2.90095400	-0.01844400	-0.00417500
S	1.27145100	-0.61743100	0.00259900
C	-2.02107300	-0.99543700	0.35031800
C	-3.24498300	-1.37707900	-0.23396100
C	-3.98061400	-0.12670800	-0.64006600
C	-1.31712500	-1.93864800	1.09901000
C	-1.78343500	-3.26495600	1.13035000
C	-2.93995200	-3.64754300	0.44815200
C	-3.70845100	-2.68002500	-0.21881700
O	-5.07123200	-0.06138900	-1.14980800
C	3.94746700	-1.00499800	-0.33982800
O	3.67754800	-2.13524500	-0.69813700
H	-4.29219200	2.72764300	-0.69137400
H	-2.46915600	4.30925200	-0.02067600
H	-0.18451700	4.74396800	0.54994300
H	2.16073200	3.99848600	0.73658400
H	3.90103900	1.85635200	0.32944800
H	-0.44842300	-1.66593100	1.68651500
H	-1.24893100	-3.99731900	1.72875100
H	-3.28330100	-4.67583200	0.49911700
H	-4.66538700	-2.92346600	-0.67216400
C	6.24280100	-1.41570000	-0.54724600
H	6.16279500	-1.72196600	-1.59149300
H	7.15655900	-0.85207500	-0.37587900
H	6.19884500	-2.29383300	0.09852800
O	5.17150800	-0.51388000	-0.21909000
K	-0.32774300	-2.87142600	-1.68636100

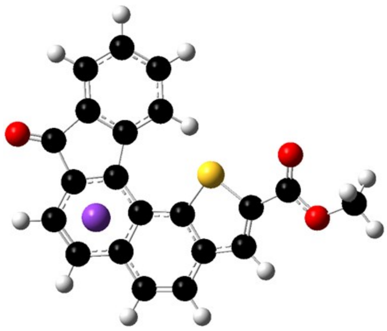

1gK⁺

M062X/6-31+G(d,p)

Symbol	*X*	*Y*	*Z*
C	-3.05274200	0.96527200	-0.25293700
C	-3.30285600	2.34083400	-0.31388800
C	-2.27029700	3.19337700	0.02216300
C	-0.96773700	2.69417300	0.28841300
C	-0.69744500	1.27847000	0.24549300
C	-1.82326400	0.41167100	0.10675800
C	0.09039100	3.62729700	0.54615000
C	1.38592500	3.22162900	0.64999000
C	1.70790300	1.85032500	0.43486700
C	0.69122100	0.90154900	0.24008800
C	3.01772400	1.29119000	0.29284900
C	2.97584300	-0.02059000	-0.06004000
S	1.36272600	-0.64463900	-0.19444300
C	-2.02463900	-1.06763600	0.27717600
C	-3.32860600	-1.38916500	-0.15114700
C	-4.03754300	-0.13731200	-0.52723700
C	-1.25918800	-2.05713500	0.88070600
C	-1.77717800	-3.35883100	0.95711000
C	-3.04004000	-3.67395000	0.46391100
C	-3.84642200	-2.66882400	-0.08141900
O	-5.15072400	0.00471200	-0.97541800
C	4.09581600	-0.95553400	-0.33157700
O	3.92537900	-2.10115300	-0.67590000
H	-4.29768200	2.69908000	-0.56333400
H	-2.43216500	4.26639300	0.07315600
H	-0.16870100	4.67695300	0.64627600
H	2.18137700	3.93446500	0.84333900
H	3.94267000	1.84021400	0.42556400
H	-0.29340200	-1.85216600	1.32291000
H	-1.17786900	-4.13271500	1.42567700
H	-3.41119000	-4.69047400	0.53680800
H	-4.86000300	-2.86635300	-0.41720800
C	6.41926300	-1.21619000	-0.38765500
H	6.42610600	-1.55069700	-1.42628000
H	7.28636100	-0.59685200	-0.17145600
H	6.38234800	-2.08232100	0.27465000
O	5.27838400	-0.37272700	-0.15351100
K	-1.20191300	1.71636100	-2.69971500

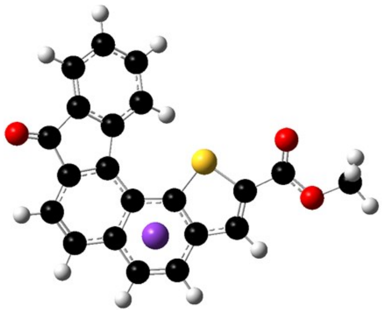

1hK⁺

M062X/6-31+G(d,p)

Symbol	X	Y	Z
C	-3.02511700	0.97811500	-0.26716100
C	-3.27526200	2.35021900	-0.32020400
C	-2.24948400	3.20227900	0.02964000
C	-0.95315100	2.69844600	0.30421000
C	-0.68528600	1.28303600	0.27134200
C	-1.80470100	0.41817600	0.10298600
C	0.11049800	3.63312700	0.55248400
C	1.40847100	3.23118100	0.66190000
C	1.72819900	1.85239200	0.47231600
C	0.70650600	0.90352900	0.28945500
C	3.03840800	1.29163400	0.32277100
C	2.99297000	-0.02119500	-0.02821200
S	1.37701100	-0.64365500	-0.15310100
C	-2.00729700	-1.06141300	0.25563000
C	-3.30117600	-1.37861900	-0.20252400
C	-4.00857500	-0.12034200	-0.56396900
C	-1.25511400	-2.05786900	0.86552500
C	-1.77180800	-3.36085900	0.91493000
C	-3.02335700	-3.67075200	0.39022900
C	-3.81915300	-2.65908100	-0.15790200
O	-5.12628000	0.02620300	-0.99573700
C	4.11303900	-0.96026500	-0.29215200
O	3.93985100	-2.10686300	-0.62938900
H	-4.26440800	2.70848700	-0.58942400
H	-2.41003100	4.27540900	0.07986900
H	-0.14878600	4.68372800	0.64731800
H	2.20347300	3.94569100	0.85229100
H	3.96483000	1.83833000	0.45751200
H	-0.30243000	-1.85741500	1.33769200
H	-1.18281400	-4.13974600	1.38832500
H	-3.39456300	-4.68857300	0.44221900
H	-4.82566600	-2.85209100	-0.51671300
C	6.43671800	-1.22558600	-0.33642200
H	6.44588200	-1.57499400	-1.37003000
H	7.30383700	-0.60442300	-0.12591400
H	6.39566500	-2.08162900	0.33850500
O	5.29546800	-0.37659600	-0.11751100
K	0.40550500	2.20508600	-2.39778800

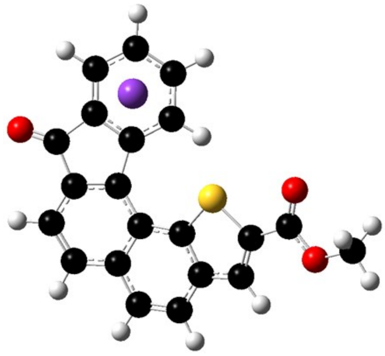

1iK+

M062X/6-31+G(d,p)

Symbol	X	Y	Z
C	-3.03033600	1.05214100	-0.35466200
C	-3.26184100	2.42675600	-0.44477500
C	-2.20344900	3.26215900	-0.17276900
C	-0.90667800	2.75046500	0.07734900
C	-0.65201400	1.33305800	0.07359600
C	-1.79283100	0.48878800	-0.03436400
C	0.15631800	3.68306200	0.29984500
C	1.44573800	3.27164800	0.43410800
C	1.75216300	1.88869800	0.29400500
C	0.73059200	0.94260400	0.11658300
C	3.05978600	1.31275500	0.22131200
C	3.01802900	-0.01839200	-0.04999500
S	1.40418800	-0.62511000	-0.22032900
C	-2.00955500	-0.98949900	0.15646100
C	-3.33115100	-1.29842200	-0.23444400
C	-4.04573700	-0.01751000	-0.54851200
C	-1.25631200	-2.00462500	0.74849700
C	-1.79329700	-3.30335700	0.82811700
C	-3.07184000	-3.60139500	0.35589600
C	-3.86950400	-2.57174000	-0.16789400
O	-5.21869700	0.10321200	-0.81623800
C	4.13343500	-0.97881100	-0.21596400
O	3.96490900	-2.15888200	-0.42029600
H	-4.24987300	2.79831700	-0.69693300
H	-2.33624300	4.33984800	-0.17996300
H	-0.09243500	4.73896100	0.33731500
H	2.24867600	3.98449800	0.59255800
H	3.98455600	1.86432100	0.34343100
H	-0.26461700	-1.82955700	1.14409500
H	-1.18202100	-4.09316800	1.25485400
H	-3.44466200	-4.61965500	0.39752000
H	-4.88598900	-2.74789900	-0.51105900
C	6.45583000	-1.24671100	-0.27141300
H	6.43269000	-1.71126700	-1.25864400
H	7.32655600	-0.60405900	-0.16415900
H	6.44555700	-2.02240000	0.49630100
O	5.32006900	-0.38270800	-0.11086700
K	-3.63559100	-1.52974300	2.78913400

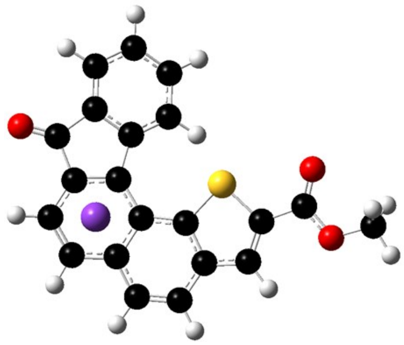

1jK⁺
M062X/6-31+G(d,p)

	Symbol *X*	*Y*	*Z*
C	2.78818500	-0.66447700	-0.74704500
C	3.10074700	-2.01792500	-0.90822400
C	2.12176700	-2.93945300	-0.59676300
C	0.80957500	-2.52363500	-0.24585500
C	0.46548300	-1.12211700	-0.18605300
C	1.54138500	-0.19181500	-0.32948000
C	-0.17690900	-3.53069700	0.01534500
C	-1.47930400	-3.20463900	0.23856000
C	-1.88219400	-1.84202900	0.15970500
C	-0.93558700	-0.82338500	-0.04665800
C	-3.22577300	-1.35551700	0.17362500
C	-3.28289200	-0.01859500	-0.06627000
S	-1.72554300	0.70226300	-0.29905100
C	1.68748300	1.28655300	-0.06970300
C	2.96729700	1.68869900	-0.50637300
C	3.72784100	0.48940600	-0.94845200
C	0.89899100	2.21557900	0.60045600
C	1.36261100	3.53428000	0.73006800
C	2.59840100	3.92800200	0.22664000
C	3.43245500	2.98418600	-0.38336000
O	4.87068600	0.41605500	-1.33251700
C	-4.47111200	0.86651000	-0.14736900
O	-4.39385400	2.05685900	-0.33828300
H	4.09011900	-2.30859500	-1.24997600
H	2.32018200	-4.00523900	-0.67012200
H	0.13548000	-4.57038700	-0.00817400
H	-2.22566600	-3.97285200	0.41506300
H	-4.10470400	-1.96968200	0.33022100
H	-0.05780100	1.95963800	1.03437600
H	0.73502100	4.25953700	1.23795400
H	2.92375100	4.95740500	0.33108500
H	4.42526300	3.24165100	-0.74019300
C	-6.80662500	0.96733700	-0.05894600
H	-6.88102600	1.44022500	-1.03947500
H	-7.61939800	0.26188200	0.09617100
H	-6.80018100	1.73453400	0.71707800
O	-5.60284300	0.18501700	0.01595100
K	2.55147700	-1.50742600	2.21992100

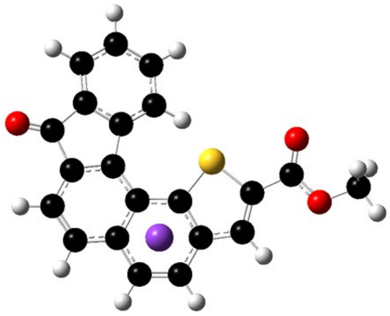

1kK⁺

M062X/6-31+G(d,p)

Symbol	X	Y	Z
C	3.87305000	-0.29444900	-0.37679700
C	4.18310500	-1.64176900	-0.55654200
C	3.20288900	-2.56635200	-0.27234600
C	1.89435600	-2.14851100	0.06986300
C	1.55525500	-0.74768000	0.15224400
C	2.63040400	0.18336100	0.03613300
C	0.90237600	-3.15704000	0.32927300
C	-0.39929700	-2.83727500	0.57958900
C	-0.80278200	-1.46904300	0.51751500
C	0.14766400	-0.45122400	0.30071100
C	-2.14680300	-0.97972400	0.53805800
C	-2.20182400	0.35722400	0.29629800
S	-0.64454200	1.07589400	0.05600100
C	2.77712900	1.66060000	0.29716800
C	4.06512100	2.05500900	-0.11750800
C	4.82101800	0.85472500	-0.56404300
C	1.98371200	2.60047400	0.94624000
C	2.45479000	3.91468800	1.08632800
C	3.70461200	4.29738400	0.61012900
C	4.53916000	3.34599400	0.01445200
O	5.95722400	0.78182000	-0.96406000
C	-3.39068300	1.24364700	0.21456500
O	-3.31010700	2.43343900	0.02564600
H	5.18031100	-1.92742200	-0.87723000
H	3.40428600	-3.63126400	-0.34237300
H	1.21291300	-4.19714000	0.28547700
H	-1.14498400	-3.60904600	0.74574500
H	-3.02728300	-1.59346000	0.68946900
H	1.01431200	2.36027300	1.36037300
H	1.82303200	4.64550500	1.58105000
H	4.03853700	5.32294800	0.72445400
H	5.54131400	3.59254900	-0.32318800
C	-5.72707600	1.34369700	0.28715300
H	-5.79241400	1.81271500	-0.69577100
H	-6.54033700	0.63782700	0.43712900
H	-5.72780400	2.11354400	1.06046700
O	-4.52228000	0.56209700	0.37524400
K	1.20666000	-1.49743300	3.05405200

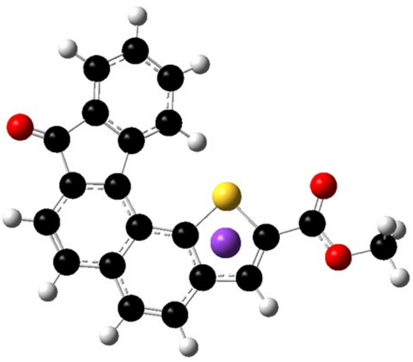

1lK$^+$
M062X/6-31+G(d,p)

Symbol	*X*	*Y*	*Z*
C	-3.03781300	0.97525800	-0.25731000
C	-3.28706700	2.34512100	-0.32537100
C	-2.23721800	3.19716800	-0.07447100
C	-0.93276100	2.69168300	0.13025600
C	-0.66906600	1.27491900	0.11761600
C	-1.79784100	0.40674200	0.02578900
C	0.12615800	3.63942400	0.32279700
C	1.42734700	3.25867000	0.41810700
C	1.74509100	1.87617700	0.27164100
C	0.72717600	0.91339200	0.12807600
C	3.06138600	1.31387900	0.18414500
C	3.03564300	-0.02836600	-0.05000100
S	1.42318200	-0.66187300	-0.18990200
C	-2.01152100	-1.07880300	0.18463800
C	-3.34460500	-1.37276900	-0.16613500
C	-4.05967400	-0.10182300	-0.45997200
C	-1.23896200	-2.11687700	0.69465500
C	-1.77438800	-3.41221000	0.76086400
C	-3.07247400	-3.68667800	0.34561500
C	-3.88496000	-2.64234700	-0.10756300
O	-5.21507700	0.05530500	-0.77125500
C	4.16720300	-0.97590600	-0.22004900
O	4.01078900	-2.17039400	-0.31332500
H	-4.29006700	2.69938200	-0.54267100
H	-2.38290600	4.27287800	-0.06350600
H	-0.14043900	4.69104100	0.36639900
H	2.22394500	3.98575300	0.53940300
H	3.98096900	1.88546700	0.25258500
H	-0.23071500	-1.97151000	1.05554700
H	-1.15464000	-4.21547200	1.14673700
H	-3.45841100	-4.69879300	0.39960900
H	-4.92088400	-2.80216100	-0.39152600
C	6.48728400	-1.19250500	-0.43916400
H	6.40019900	-1.71043000	-1.39526500
H	7.34342200	-0.52262600	-0.43565900
H	6.55660900	-1.92355500	0.36780000
O	5.33856800	-0.34679100	-0.24001300
K	1.69007100	0.29117700	2.92663000

2.2 Determination of interaction energies

The binding energy is defined as the energy gain related to the formation of the complex. The energy obtained is positive if the interaction is favourable. This can be explained by the equation (1):

$$Einteraction = E(receptor)ZPE + E(cation) - E(receptor{:}cation)ZPE \quad (1)$$

where :

- E(receptor)ZPE is the energy of the receptor corrected by the Zero Point Energy

- E(cation) is the is the energy of the cation

- E(receptor:cation) ZPE is the energy of the complex corrected by the Zero Point Energy

All these energies, expressed in kJ/mol, are obtained from optimized structures at the M06-2X/6-31G+(d,p) level, and are corrected by the Zero Point Energy. Energies then refined with a bigger basis set. BSSE not included as generally very small at these levels.

Table S2. *Computed binding energies*

Structure	BE_1 [a]	BE_2 [b]	Structure	BE_1 [a]	BE_2 [b]
**1aK+**	75.5	77.9	**1gK+**	83.6	87.5
**1bK+**	59.0	60.3	**1hK+**	81.0	84.9
**1cK+**	115.1	117.9	**1iK+**	79.0	83.9
**1dK+**	86.3	90.3	**1jK+**	78.7	83.3
**1eK+**	116.3	118.2	**1kK+**	74.4	78.0
**1fK+**	98.4	101.6	**1lK+**	67.5	71.5

a : M06-2X/6-31+G(d,p)

b. M06-2X/6-311++G(2df,2pd)//M06-2X/6-31+G(d,p)

3. Photophysical analysis and procedures

3.1 General practical analysis procedure

A solution of **1** in THF was prepared (3,6µM). 3mL of this stock solution was added to a quartz cuvette. A second solution containing **1** (3.6µM) and KTBPB (1,63mM) was prepared. Aliquots of this second solution were added to the quartz cuvette. After each addition, a UV spectrum was recorded.

Binding constant was determined using SPECFIT/32 ™ Global Analysis System software. [5,6] This software allows global analysis of equilibrium and kinetic systems with Expanded SVD and nonlinear regression modeling by the Levenberg-Marquardt method.

3.2 Time dependant DFT analysis of 1

In order to attribute bands observed on **1**, theoretical UV spectra was calculated.

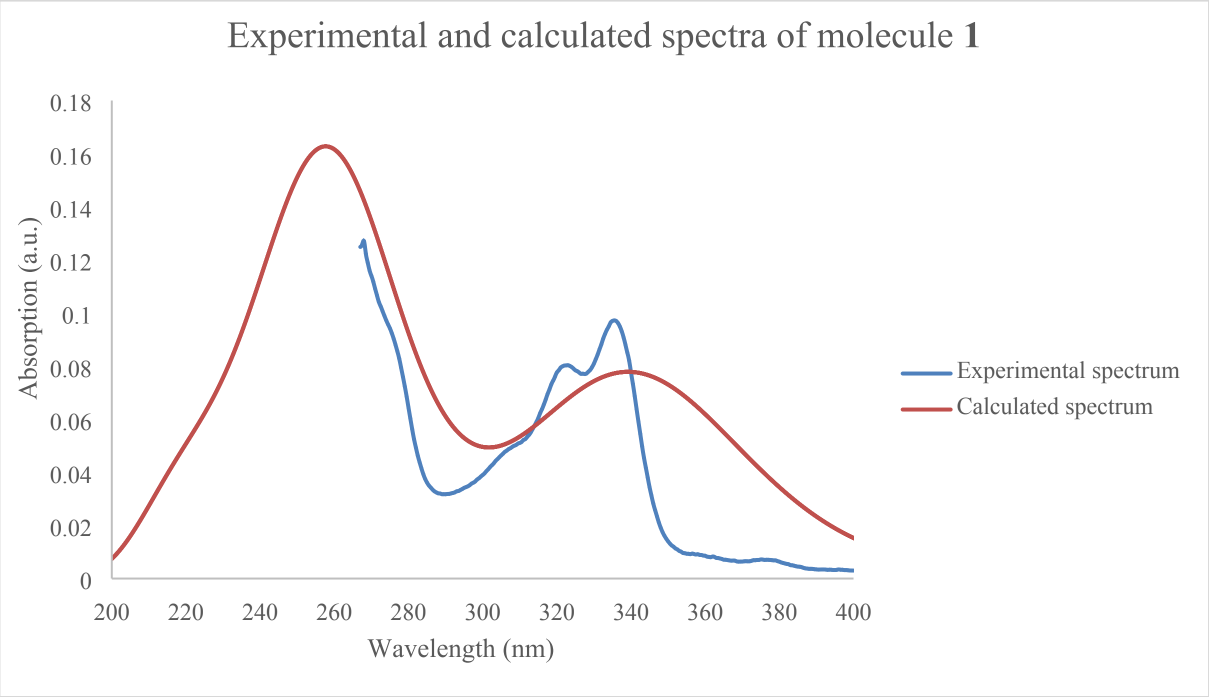

Figure S2. Experimental and calculated spectra of molecule **1**

No.	*Wavelength*	*Osc.*	*Major contribs*

	(nm)	*Strength*	
1	*455.322045583*	*0.016*	*HOMO->LUMO (96%)*
2	*379.16814891*	*0.0014*	*H-4->LUMO (19%), H-3->LUMO (63%), H-1->LUMO (10%)*
3	*374.619872529*	*0.0072*	*H-1->LUMO (59%), HOMO->L+1 (25%)*
4	***342.980975994***	***0.5216***	***H-1->LUMO (28%), HOMO->L+1 (69%)***
5	*325.417829428*	*0.0071*	*H-2->LUMO (87%)*
6	***305.041685354***	***0.1323***	***H-1->L+1 (72%)***
7	*300.065812368*	*0.0103*	*H-4->LUMO (56%), H-3->LUMO (21%), H-1->L+1 (10%)*
8	*269.994540651*	*0.0458*	*H-2->L+1 (73%), HOMO->L+2 (20%)*
9	***265.014092451***	***0.4036***	***H-5->LUMO (13%), HOMO->L+2 (42%), HOMO->L+3 (17%)***
10	***263.504618321***	***0.3424***	***H-2->L+1 (10%), HOMO->L+2 (17%), HOMO->L+3 (45%)***
11	*259.278096598*	*0.0196*	*H-4->L+1 (18%), H-3->L+1 (60%), HOMO->L+3 (11%)*
12	*252.529060864*	*0.0871*	*H-6->L+1 (12%), H-5->LUMO (34%), H-4->L+1 (21%), H-3->L+1 (13%)*
13	*250.761873293*	*0.1042*	*H-6->LUMO (13%), H-6->L+1 (47%), H-5->LUMO (19%)*
14	***249.08928782***	***0.1981***	***H-5->LUMO (13%), H-4->L+1 (45%)***
15	***246.803473629***	***0.0116***	***H-1->L+2 (66%)***
16	*241.543333357*	*0.1621*	*H-7->LUMO (75%), H-1->L+2 (12%)*
17	*235.931177356*	*0.066*	*H-5->L+1 (14%), H-1->L+3 (70%)*
18	*233.905959726*	*0.0006*	*H-6->LUMO (80%), H-6->L+1 (16%)*
19	*228.121790271*	*0.0875*	*H-2->L+2 (14%), HOMO->L+4 (65%)*
20	*224.588702132*	*0.0587*	*H-5->L+1 (69%), H-1->L+3 (14%)*
21	*221.66158868*	*0.0222*	*H-2->L+2 (19%), HOMO->L+5 (56%)*
22	*220.734200381*	*0.0442*	*H-2->L+2 (43%), HOMO->L+5 (24%)*
23	*218.178318426*	*0.0025*	*H-4->L+2 (19%), H-3->L+2 (65%)*
24	*214.083283856*	*0.1154*	*H-2->L+3 (45%), H-1->L+4 (29%)*

Figure S3. Calculated transitions (major transitions in bold)

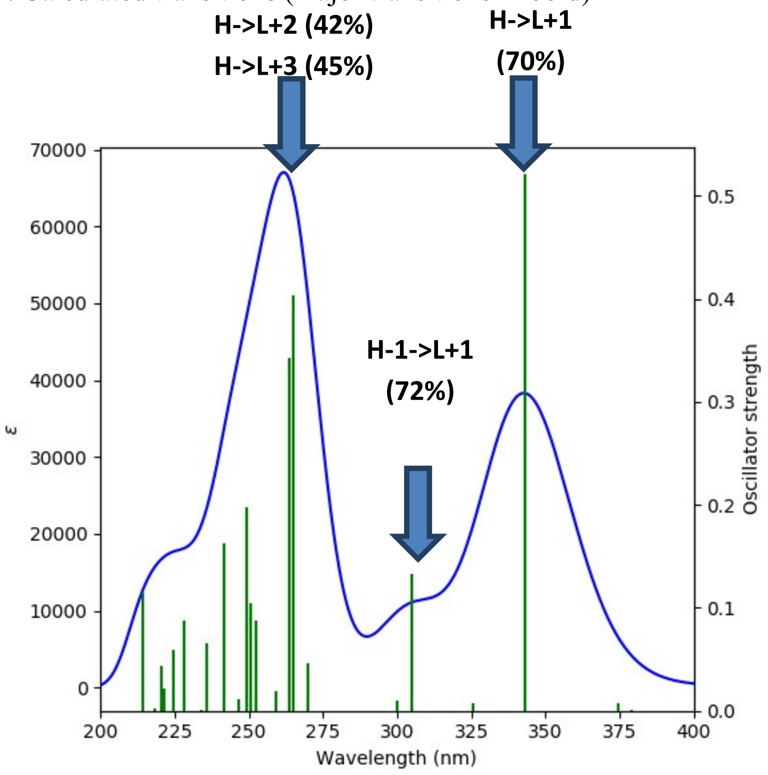

Figure S4. Main electronic transitions identified and calculated

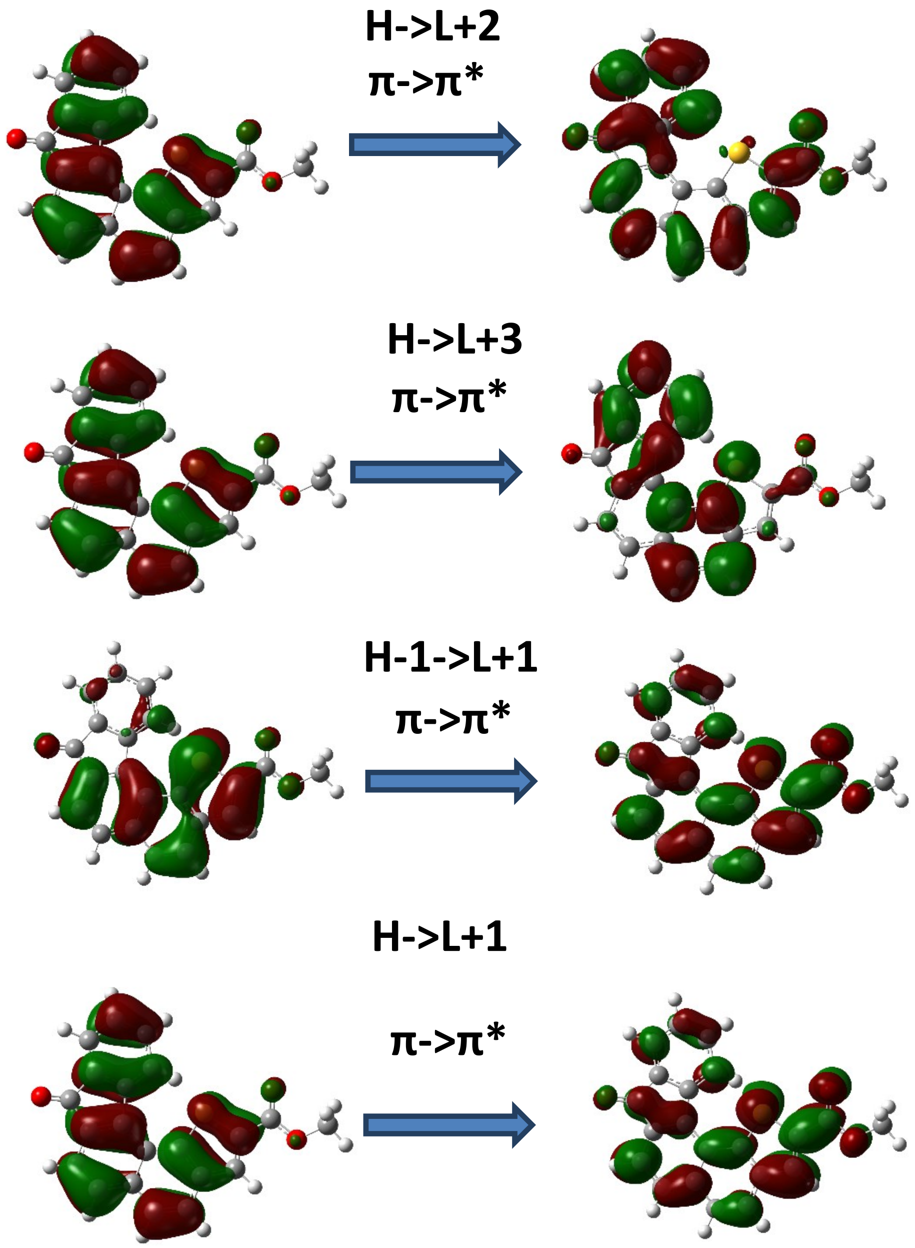

Figure S5. Nature of molecular orbitals involved in the main transitions

4. Mass spectrometry

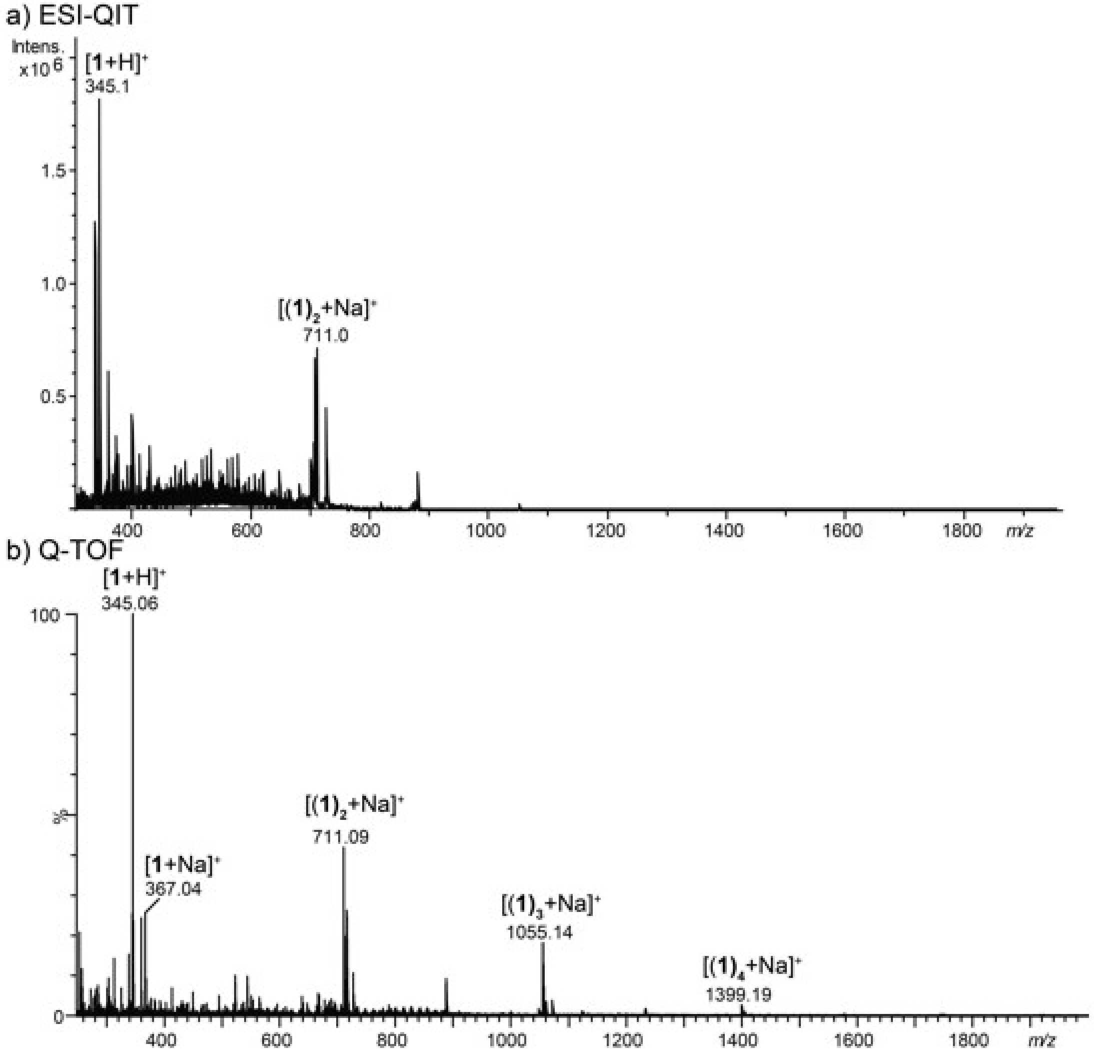

Figure S6. *MS spectra obtained from a methanolic solution of **1** analyzed on **a)** a QIT and **b)** a Q-TOF mass spectrometer, equipped with electrospray sources.*

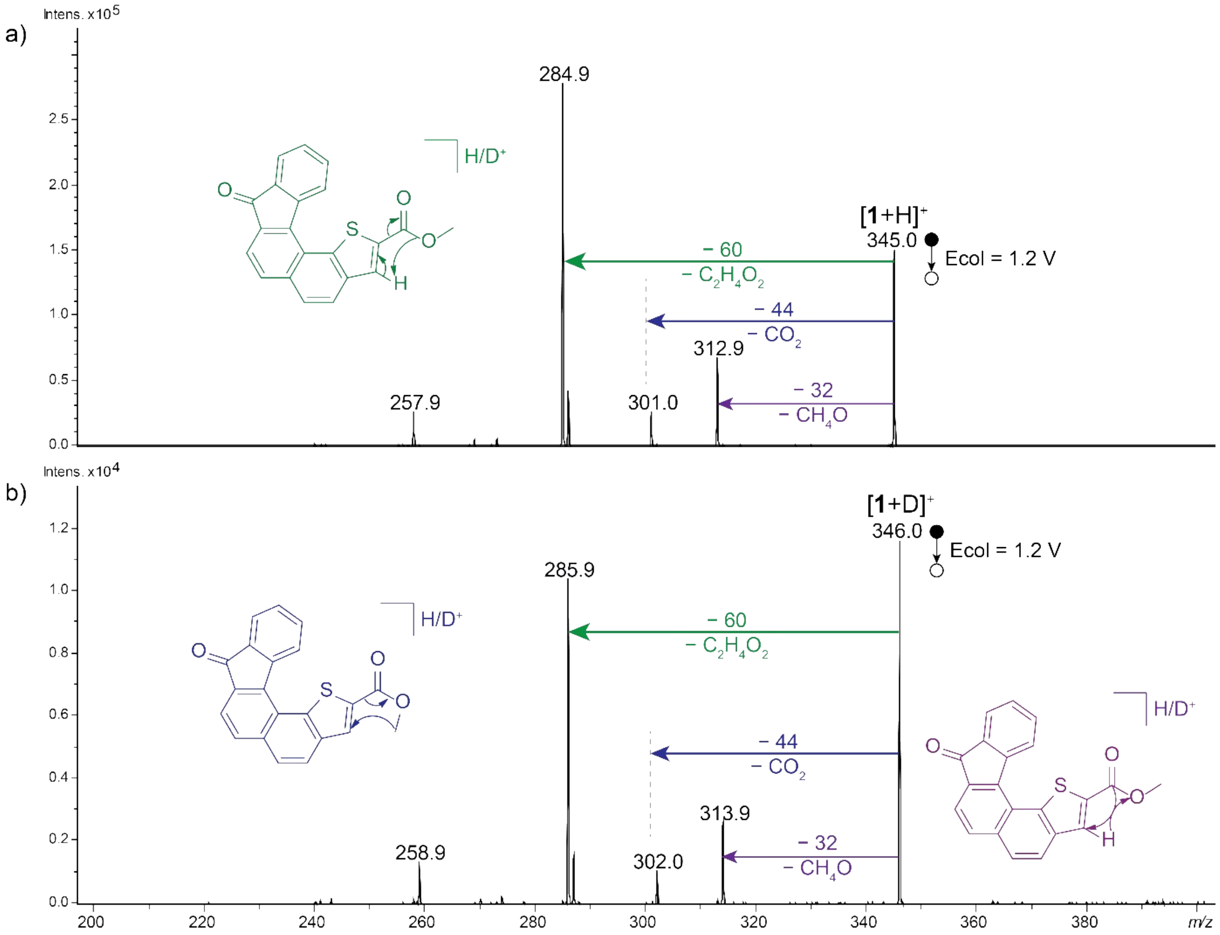

Figure S7. *MS/MS spectra of **a)** [1+H]+ at m/z 345 in $CH_3OH/CHCl_3$ 8:2 and **b)** [1+D]+ at m/z 346 in $CH_3OD/CDCl_3$ 8:2 from an ESI-QIT mass spectrometer.*

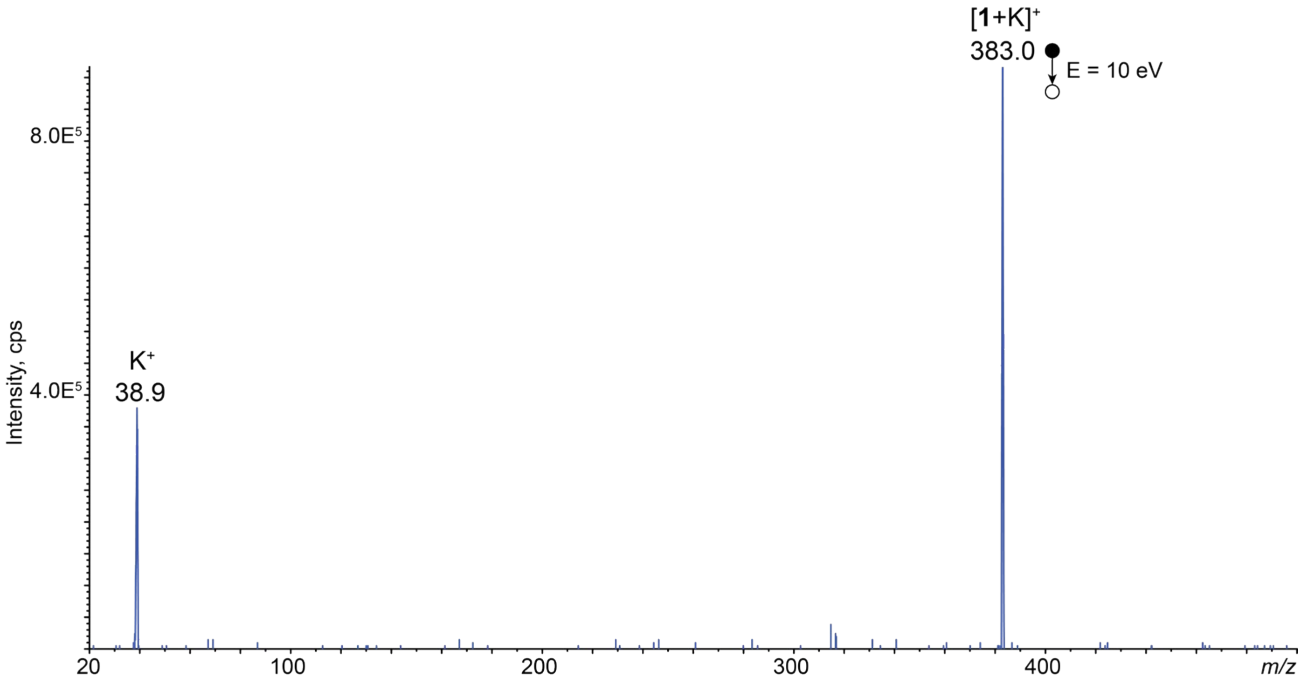

Figure S8. *MS/MS spectra of [**1**+K]$^+$ (m/z 383) recorded with a triple-quadrupole instrument (API 2000; ABSciex). The only noticeable fragment ion is K$^+$.*

5. IRMPD data

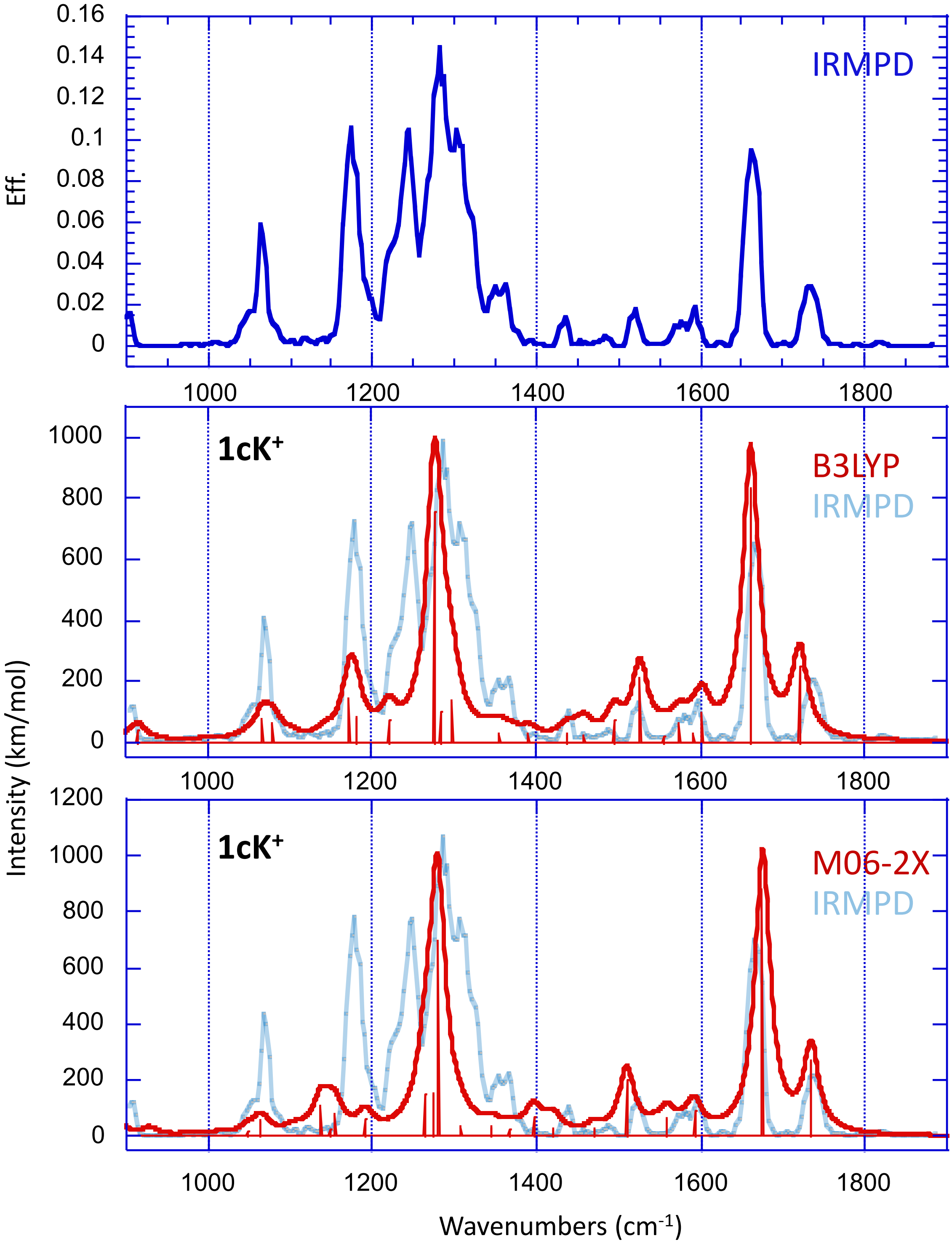

Figure S9. *Comparison between the experimental IRMPD spectrum of the [**1**+K]⁺ complex , and the spectra computed for the* ***1cK⁺*** *structure, either with the B3LYP or the M06-2X functional and the 6-31+G(d,p) standard basis set (scaling factors of 0.974 and 0.940, respectively).*

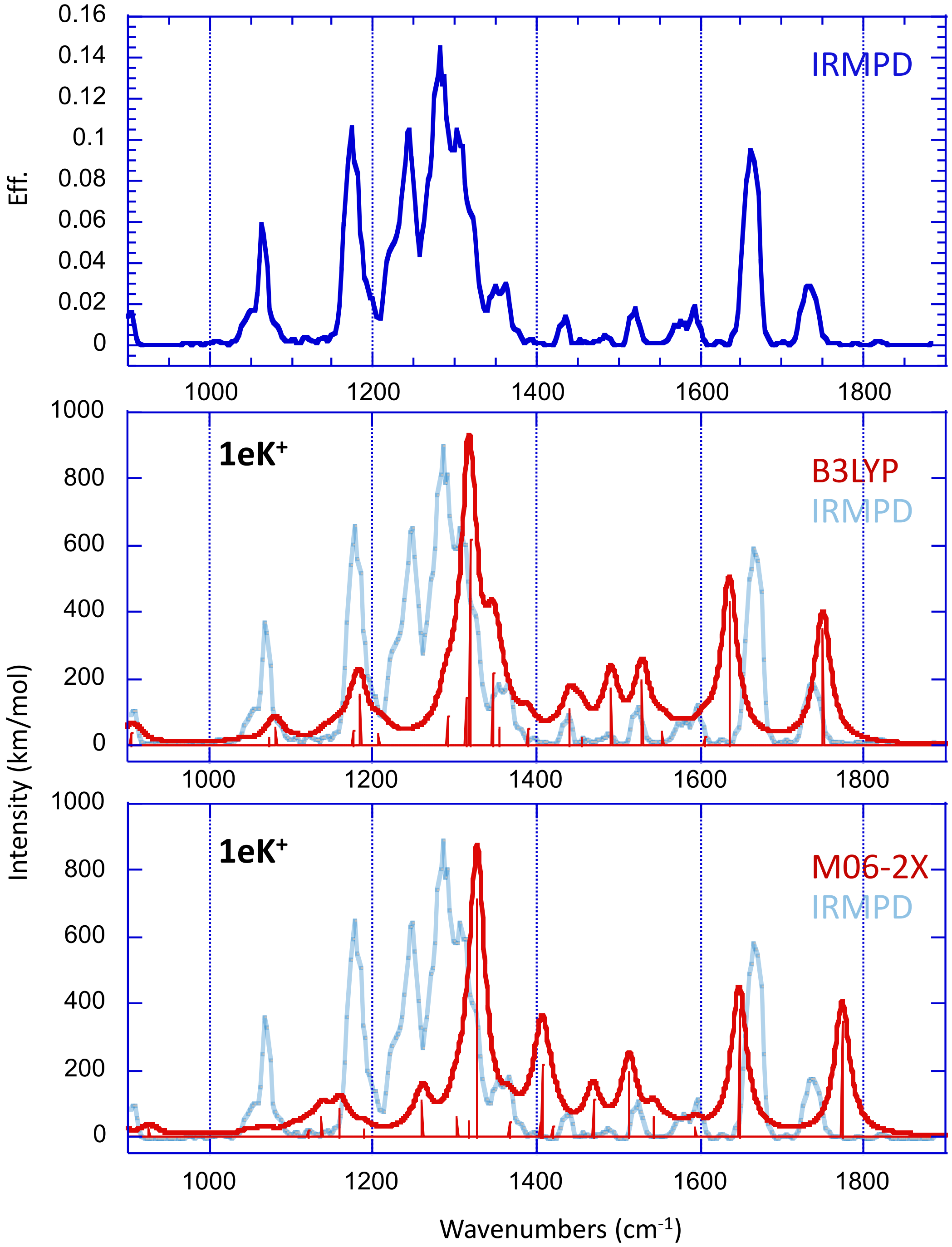

Figure S10. *Comparison between the experimental IRMPD spectrum of the [**1**+K]+ complex , and the spectra computed for the **1eK+** structure, either with the B3LYP or the M06-2X functional and the 6-31+G(d,p) (scaling factors of 0.974 and 0.940, respectively).*

6. BLM experiments

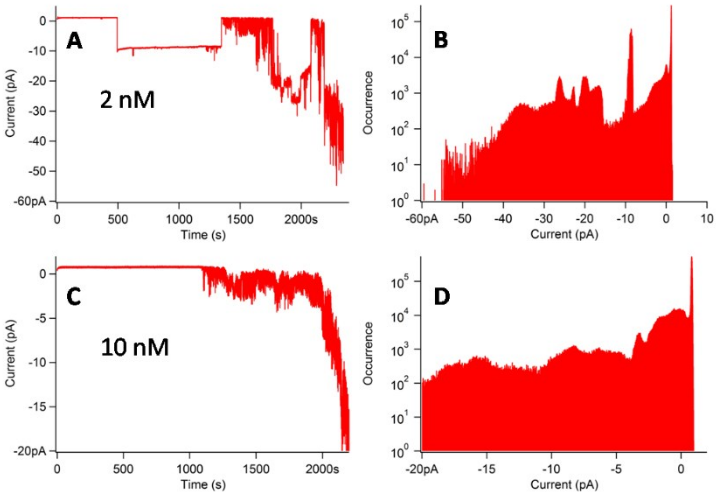

Figure S11 (A/C)*-Current-time traces for an applied voltage of -100 mV, in presence of* ***3*** *([****3****] = 2 or 10 nM-[KCl] = 1 M)* ***(B/D)****-Corresponding current distributions*

7. Synthetic procedures and characterization data

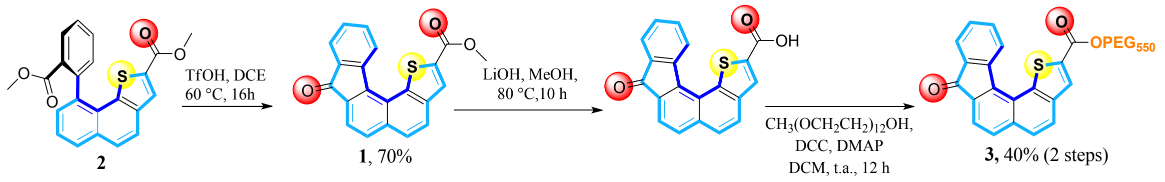

7.1 Preparation of methyl 8-oxo-8H-indeno[2',1':7,8]naphtho[1,2-b]thiophene-2-carboxylate 1

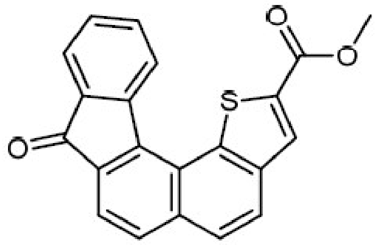

Chemical Formula: $C_{21}H_{12}O_3S$
Molecular Weight: 344.38 g.mol-1
Trifluoromethanesulfonic acid (140 µL, 1.6 mmol, 12 eq.) was added dropwise to a solution of methyl 9-(2-(methoxycarbonyl)phenyl)naphtho[1,2-b]thiophene-2-carboxylate (50mg, 0.13 mmol, 1 eq.) in dry 1,2-dichloroethane (8.5 mL) at room temperature. After being stirred at 60 °C overnight, the reaction was slowly poured over an aqueous solution of saturated $NaHCO_3$. The mixture was then extracted with methylene chloride and the combined organic layers were washed with brine, dried over anhydrous $MgSO_4$, filtered and the solvent was removed under reduced pressure. The crude product was purified by flash chromatography on silica gel eluting with methylene chloride to afford methyl 8-oxo-8H-indeno[2',1':7,8]naphtho[1,2-b]thiophene-2-carboxylate (31.3 mg, 0.091 mmol, 70 %) as an orange powder.

^{1}H NMR (300 MHz, $CDCl_3$, 25°C, TMS) δ 8.86 (d, J = 7.5 Hz, 1H), 8.15 (s, 1H), 7.82–7.90 (m, 3H), 7.74 (d, J = 7.9 Hz, 2H), 7.60 (t, J = 7.5 Hz, 1H), 7.37 (t, J = 7.3 Hz, 1H), 4.01 (s, 3H).
^{13}C NMR (75 MHz, THF-*d8*, 25°C, TMS) δ = 192.7, 162.6, 154.7, 145.6, 140.6, 139.6, 138.5, 135.4, 135.2, 135.2, 132.5, 132.3, 131.2, 130.0, 128.7, 126.0, 125.5, 125.2, 16.7, 121.0, 52.5 ppm.
HRMS (ESI+-TOF) m/z calcd for $C_{21}H_{13}O_3S$ $[M+H]^+$ 345.0585, found : 345.0582.
Mp 254 °C

Data were in accordance with literature. [7]

7.2 Preparation of 8-Oxo-8H-indeno[2',1':7,8]naphtho[1,2-b]thiophene-2-carboxylic acid

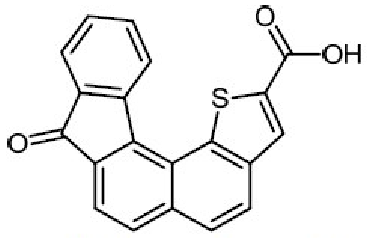

Chemical Formula: $C_{20}H_{10}O_3S$
Molecular Weight: 330.36 g.mol-1
Methyl 8-oxo-8H-indeno[2',1':7,8]naphtho[1,2-b]thiophene-2-carboxylate (50 mg, 0.145 mmol) was dissolved in methanol (3.3 mL) and a solution of LiOH (1*M*, 1.5 mL) was added. The mixture was heated under stirring at 85°C overnight. The reaction mixture was transferred to a separatory funnel, rinsed with ethyl acetate and a solution of HCl (1*M*) was added to the filtrate drop by drop until pH four. The organic layer was then washed with water and brine and dried over anhydrous $MgSO_4$. The organic layer was filtered and concentrated under vacuum to give 8-oxo-8H-indeno[2',1':7,8]naphtho[1,2-b]thiophene-2- carboxylic acid (38 mg, 0.12 mmol 79 %) as a red solid which was used in the next step without further purification.

^{1}H NMR (300 MHz, DMSO-*d6*, 25°C, TMS) *δ* 8.78–8.81 (d, *J* = 7.5 Hz, 1H), 8.26 (s, 1H), 8.06–8.13 (t, *J*= 7 Hz, 2H), 7.92–7.97 (d, *J* = 9 Hz, 1H), 7.68–7.80 (m, 3H), 7.41–7.48 (t, *J* = 7 Hz, 1H), 6.57 (s, 1H) ppm.
^{13}C NMR (75 MHz, DMSO-*d6*, 25°C, TMS) *δ* 162.9, 144.2, 139.9, 138.4, 135.2, 133.7, 131.9, 130.4, 129.7, 127.7, 125.9, 124.3, 120.3, 40.3, 40.1, 39.8, 39.5, 39.2, 38.9, 38.7 ppm.
HRMS (ESI+-TOF) *m*/z calcd for C20H11O3S $[M+H]^+$ 331.0429 ; found : 331.0421.

7.3 Preparation of 2,5,8,11,14,17,20,23,26,29,32,35-Dodecaoxaheptatriacontan-37-yl 8-oxo-8H-indeno[2',1':7,8]naphtho[1,2-b]thiophene-2-carboxylate 3

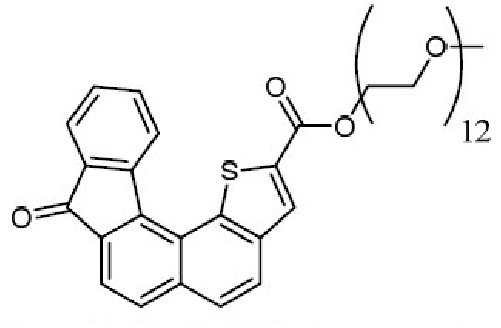

Chemical Formula: $C_{45}H_{60}O_{15}S$
Molecular Weight: 873.01 g.mol-1
8-oxo-8H-indeno[2',1':7,8]naphtho[1,2-b]thiophene-2-carboxylic acid (30 mg, 91µmol) was dissolved in dichloromethane (1 mL) and DCC (23 mg, 1.1 eq.) and DMAP (6 mg, 0.5 eq.) were added under argon. The mPEG550 (61 mg, 50µL, 0.1 mmol) was added and left stirring at 25°C overnight. The mixture was rinsed with dichloromethane and filtered through cotton to remove the urea formed. The filtrate was then extracted with water and dichloromethane five times. The organic layers were dried over sulfate magnesium, filtered and concentrated under vacuum. The product was purified firstly by column chromatography on silica gel eluting with a mixture of methylene chloride/methanol (95:5) and secondly on a preparative TLC eluting with ethyl acetate/methanol to give 2,5,8,11,14,17,20,23,26,29,32,35-dodecaoxaheptatriacontan-37-yl 8-oxo-8H-indeno[2',1':7,8]naphtho[1,2-b]thiophene-2-carboxylate (40 mg, 45.8 µmol, 51 %) as a yellow oil.
^{1}H NMR (300 MHz, $CDCl_3$, 25°C, TMS) *δ* 8.83–8.86 (d, *J* = 8 Hz, 1H), 8.16 (s, 1H), 7.72–7.89 (m, 5H), 7.56–7.61 (t, *J* = 7 Hz, 1H), 7.33–7.38 (t, *J* = 7 Hz, 1H), 4.54–4.57 (t, *J* = 4.5 Hz, 2H), 3.87–3.90 (t, *J* = 4.7 Hz, 2H), 3.54–3.75 (m, 44H), 3.38 (s, 3H).
^{13}C NMR (75 MHz, CDCl3) *δ* 193.4, 162.3, 144.9, 142.8, 139.4, 138.7, 138.3, 134.8, 134.6, 134.3, 131.7, 131.4, 130.7, 129.3, 127.8, 125.3, 124.9, 124.6, 124.5, 120.8, 77.5, 77.3, 77.1, 76.6, 71.9, 70.8, 70.6, 69.1, 64.9, 59.1, 29.7.
HRMS (ESI+-TOF) *m*/*z* calcd for [M+H+] $C_{45}H_{60}O_{15}S$: 873.3731; found : 873.3712.

7.4 ^{1}H NMR, ^{13}C NMR and mass spectra of 2,5,8,11,14,17,20,23,26,29,32,35-Dodecaoxa heptatriacontan-37-yl 8-oxo-8H-indeno[2',1':7,8]naphtho[1,2-b]thiophene-2-carboxylate 3

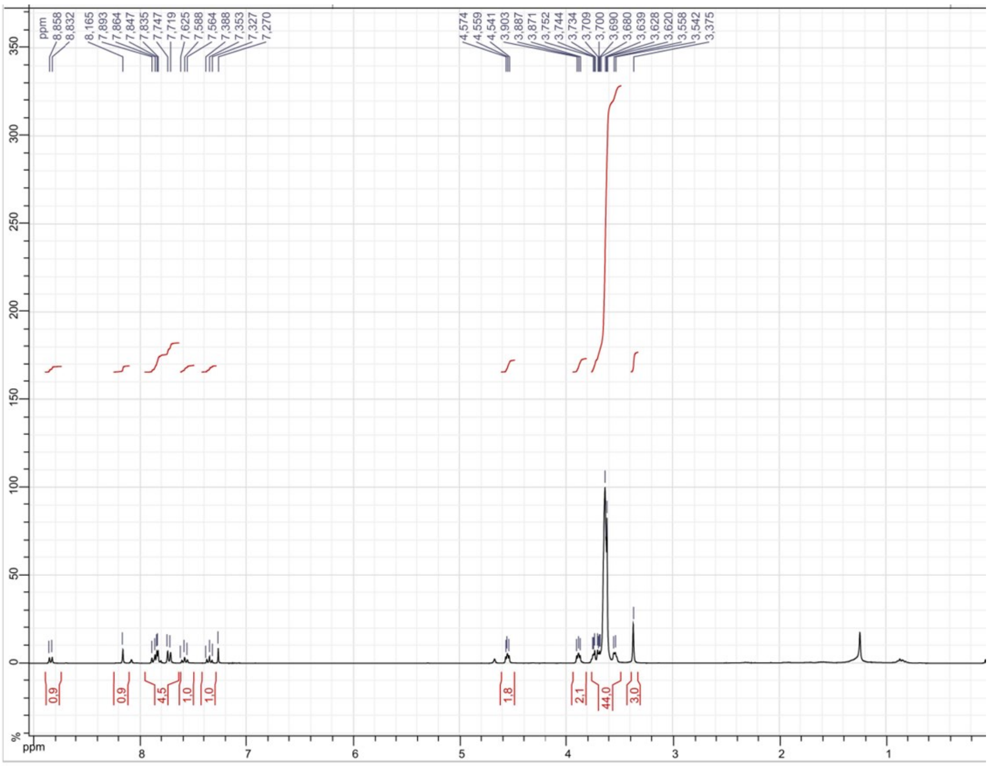

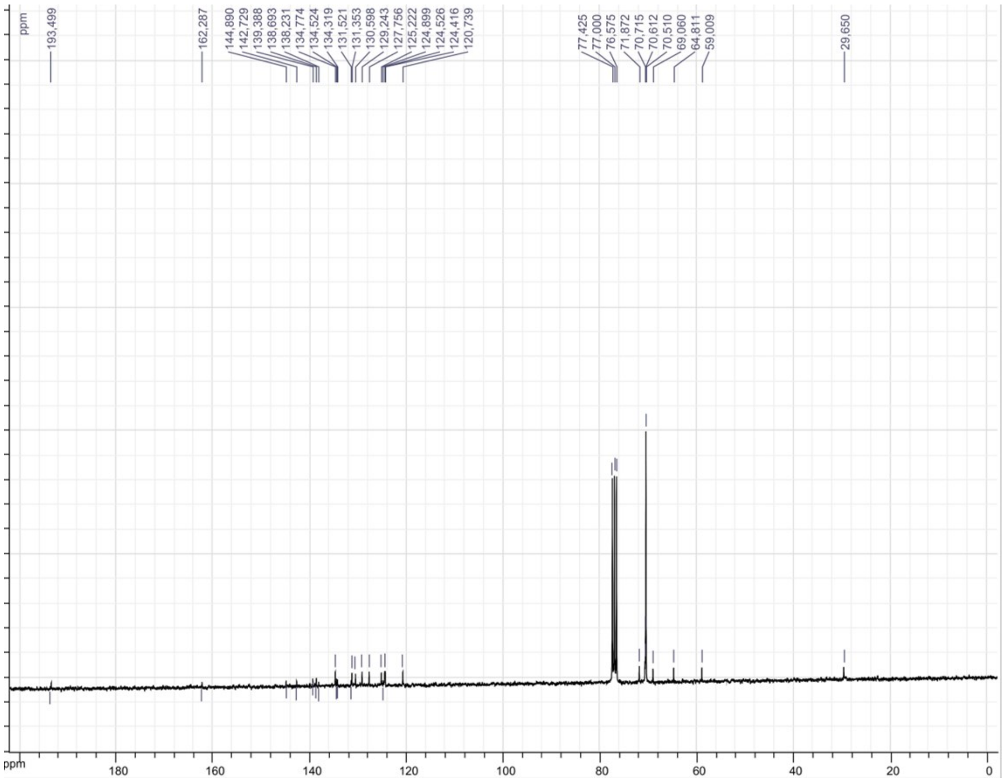

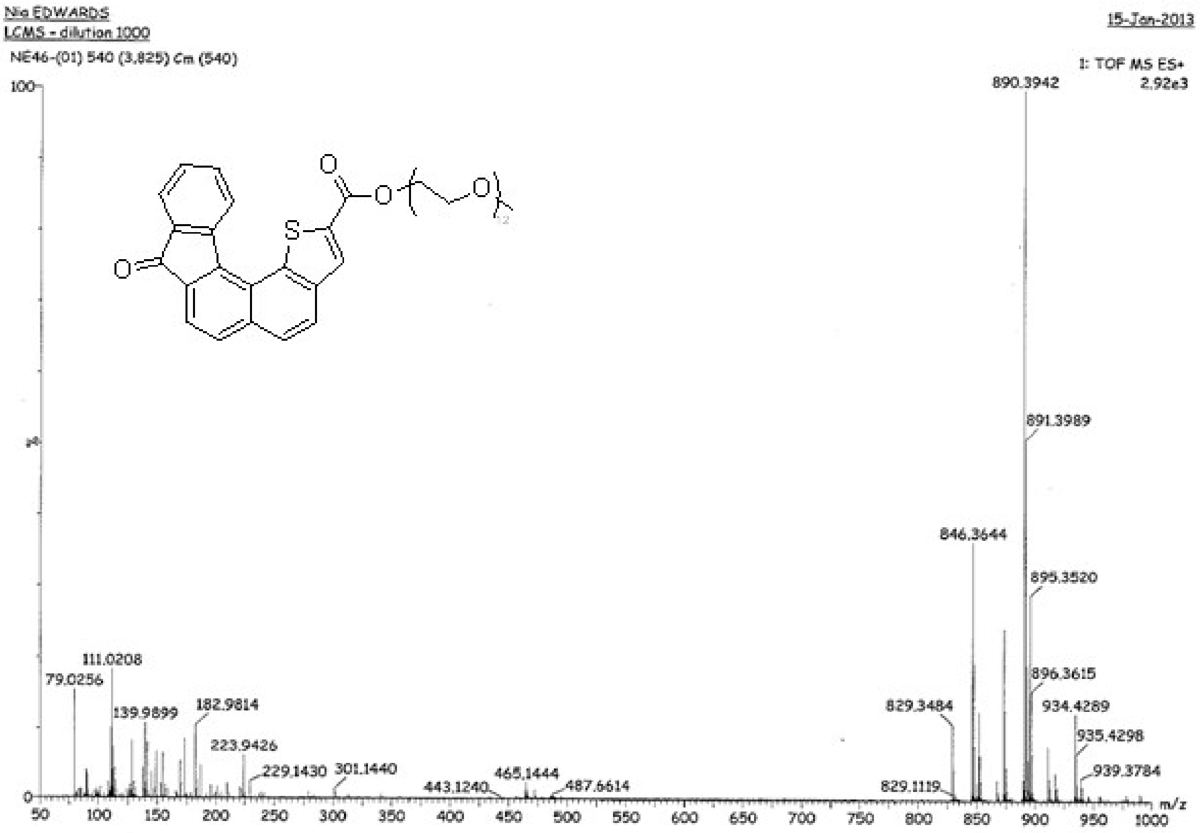

8. Bibliography

1 H. E. Gottlieb, V. Kotlyar and A. Nudelman, *J. Org. Chem.*, 1997, **62**, 7512–7515.

2 Henry S. Rzepa, Script for creating an NCI surface as a JVXL compressed file from a (Gaussian) cube of total electron density, https://www.ch.ic.ac.uk/rzepa/cub2nci/, (accessed 3 September 2019).

3 J. Contreras-García, E. R. Johnson, S. Keinan, R. Chaudret, J. P. Piquemal, D. N. Beratan and W. Yang, *J. Chem. Theory Comput.*, 2011, **7**, 625–632.

4 N. M. O'Boyle, A. L. Tenderholt, K. M. Langner. *J. Comp. Chem.*, 2008, **29**, 839-845.

5 H. Gampp, M. Maeder, C. J. Meyer, A. D. Zuberbühler, *Talenta* 1985, **32**, 95-101.

6 H. Gampp, M. Maeder, C. J. Meyer, A. D. Zuberbühler, *Talenta* 1985, **32**, 257-264.

7 A. Souibgui, A. Gaucher, J. Marrot, F. Aloui, F. Mahuteau-Betzer, B. Ben Hassine and D. Prim, *European J. Org. Chem.*, 2013, **2013**, 4515–4522.
